# Integrative Model of Oxidative Stress Adaptation in the Fungal Pathogen *Candida albicans*

**DOI:** 10.1371/journal.pone.0137750

**Published:** 2015-09-14

**Authors:** Chandrasekaran Komalapriya, Despoina Kaloriti, Anna T. Tillmann, Zhikang Yin, Carmen Herrero-de-Dios, Mette D. Jacobsen, Rodrigo C. Belmonte, Gary Cameron, Ken Haynes, Celso Grebogi, Alessandro P. S. de Moura, Neil A. R. Gow, Marco Thiel, Janet Quinn, Alistair J. P. Brown, M. Carmen Romano

**Affiliations:** 1 Institute of Complex Systems and Mathematical Biology, University of Aberdeen, Aberdeen, United Kingdom; 2 School of Medical Sciences, University of Aberdeen, Foresterhill, Aberdeen, United Kingdom; 3 School of Medicine and Dentistry, University of Aberdeen, Foresterhill, Aberdeen, United Kingdom; 4 College of Life and Environmental Sciences, University of Exeter, Exeter, United Kingdom; 5 Institute for Cell and Molecular Biosciences, University of Newcastle, Newcastle upon Tyne, United Kingdom; Louisiana State University, UNITED STATES

## Abstract

The major fungal pathogen of humans, *Candida albicans*, mounts robust responses to oxidative stress that are critical for its virulence. These responses counteract the reactive oxygen species (ROS) that are generated by host immune cells in an attempt to kill the invading fungus. Knowledge of the dynamical processes that instigate *C*. *albicans* oxidative stress responses is required for a proper understanding of fungus-host interactions. Therefore, we have adopted an interdisciplinary approach to explore the dynamical responses of *C*. *albicans* to hydrogen peroxide (H_2_O_2_). Our deterministic mathematical model integrates two major oxidative stress signalling pathways (Cap1 and Hog1 pathways) with the three major antioxidant systems (catalase, glutathione and thioredoxin systems) and the pentose phosphate pathway, which provides reducing equivalents required for oxidative stress adaptation. The model encapsulates existing knowledge of these systems with new genomic, proteomic, transcriptomic, molecular and cellular datasets. Our integrative approach predicts the existence of alternative states for the key regulators Cap1 and Hog1, thereby suggesting novel regulatory behaviours during oxidative stress. The model reproduces both existing and new experimental observations under a variety of scenarios. Time- and dose-dependent predictions of the oxidative stress responses for both wild type and mutant cells have highlighted the different temporal contributions of the various antioxidant systems during oxidative stress adaptation, indicating that catalase plays a critical role immediately following stress imposition. This is the first model to encapsulate the dynamics of the transcriptional response alongside the redox kinetics of the major antioxidant systems during H_2_O_2_ stress in *C*. *albicans*.

## Introduction

*Candida albicans* is a major opportunistic fungal pathogen of humans. It normally exists as a harmless commensal of the skin, oral cavity and gastrointestinal and urogenital tracts in about 70% of individuals. However, *C*. *albicans* frequently causes oral and vaginal infections (thrush) which can become recurrent in about 5% of patients. In immunocompromised patients, however, *C*. *albicans* can cause life-threatening invasive systemic infections [1], and the mortality rates associated with systemic candidiasis in neonatal infants, chemotherapy and transplant patients is estimated to be between 45% and 75% [2]. With the increase in the global burden of immunocompromised patients, and with the increasing proportion of elderly individuals, candidiasis has become a major healthcare issue [3]. In the last two decades substantial progress has been made in our understanding of *C*. *albicans* genetics, molecular biology and virulence mechanisms, and consequently it has become recognised as a model fungal pathogen [4].

To flourish within its human host, *C*. *albicans* must avoid immune recognition or survive the attacks of phagocytes, which are programmed to phagocytose and kill invading microorganisms. Phagocytic killing is achieved via a battery of toxic chemicals, including reactive oxygen species (ROS) such as H_2_O_2_, which exert both cytostatic and toxic effects upon the engulfed microbe [5]. The ability of *C*. *albicans* to survive exposure to the potent ROS employed by phagocytes is highly significant in the context of host-pathogen interactions, because the inactivation of oxidative stress responses attenuates the virulence of this fungus and its ability to counteract phagocytic killing [2,6,7]. Furthermore, mounting evidence suggests that oxidative stress responses are closely interlinked with other virulence attributes of *C*. *albicans*, such as morphogenesis and metabolic flexibility [8,9]. Clearly, the elaboration of oxidative stress response mechanisms is important for a proper understanding of fungal pathogenicity.

At relatively low levels ROS can act as a second-messenger in fungal cells, promoting growth and development [10]. However at higher levels, ROS exert cytotoxic effects by altering cellular redox homeostasis and by damaging DNA, proteins and lipids. Like other yeasts, *C*. *albicans* responds by activating ROS detoxification mechanisms such as catalase, by inducing major antioxidant systems such as the glutathione and thioredoxin systems, and by repairing the oxidative damage caused by the ROS to protein thiol groups, for example, via the glutathione and thioredoxin systems [7,11].

Our understanding of these oxidative stress responses in *C*. *albicans* lags behind those in the model yeasts *Saccharomyces cerevisiae* and *Schizosaccharomyces pombe*. Nevertheless, it is clear that *C*. *albicans* is more resistant to oxidative stress than these benign model yeasts, and yet during evolution, *C*. *albicans* has retained similar oxidative stress signalling modules and antioxidant systems required for adaptation to ROS exposure [11,12,13,14,15,16]. Cap1 is orthologous to AP-1-like transcription factors in *S*. *cerevisiae* (Yap1) and *S*. *pombe* (Pap1), and is largely responsible for the activation of oxidative stress genes in *C*. *albicans*. Following ROS exposure, Cap1 becomes oxidized at evolutionarily conserved redox-sensitive cysteine residues, and this transcriptional activator then accumulates in the nucleus, where it activates genes that carry Yap1 Response Elements in their promoters [17,18,19,20]. In response to oxidative stress, the Hog1 mitogen activated protein kinase (MAPK) becomes activated in a thioredoxin (Trx1) and peroxidase (Tsa1) dependent manner [21]. Hog1 contributes to oxidative stress resistance in *C*. *albicans*, mediating adaptation at both transcriptional and post-transcriptional levels [14,15,19].

Three main antioxidant systems mediate H_2_O_2_ detoxification in *C*. *albicans* [7,22]. Firstly, H_2_O_2_ dismutation is catalysed by catalase, which is induced by transcriptional activation of the single catalase gene in *C*. *albicans*, *CAT1* [15,20]. Secondly, H_2_O_2_ detoxification is mediated by the glutathione system. Glutathione peroxidases (Gpx1-3) catalyse the breakdown of H_2_O_2_ using the tripeptide glutathione (GSH: L-gamma-glutamyl-L-cysteinyl-glycine) as reductant. GSH becomes oxidised to form GSSG, which is then reduced via glutathione reductase (Glr1) using NADPH. The glutathione system also repairs oxidatively damaged protein thiols through the glutaredoxins (Grx1, Grx3, Ttr1, orf19.4150). Thirdly, the thioredoxin system detoxifies H_2_O_2_ via peroxiredoxin (Tsa1), which uses the reductant thioredoxin (Trx1). Oxidised thioredoxin is then reduced using NADPH via the action of thioredoxin reductase (Trr1). Following exposure to H_2_O_2_, *C*. *albicans* induces the expression of key elements of all three antioxidant systems as well as NADPH production via the pentose phosphate pathway (PPP) [19]. This transcriptional induction is mediated primarily by Cap1, with some contribution from Hog1 signalling [19].

While significant progress has been made with the molecular dissection of oxidative stress responses in *C*. *albicans*, the dynamics of Cap1 and Hog1 signalling and the various antioxidant systems are less well characterised. In general, experimental studies have addressed the roles of individual stresses, signalling cascades or antioxidant systems [14,15,19,21–23]. Modelling studies that integrate the collective action of the various oxidative stress response mechanisms would enhance our understanding of the dynamics of these components and their relative contributions to the system as a whole. However, with the exception of the model of oxidative stress in mammalian cells by Adimora *et al* [24], which did not include transcriptional regulation; there has been no comprehensive study that integrates the main anti-oxidant systems in any model organism.

In this study we have taken a systems level approach to unveil the emerging mechanisms by which the major signalling cascades and anti-oxidant systems mediate oxidative stress adaptation in *C*. *albicans*. We have constructed and experimentally validated a comprehensive model that, for the first time, integrates Cap1 and Hog1 signalling with the catalase, glutathione and thioredoxin antioxidant systems, and NADPH production via the pentose phosphate pathway. Furthermore, this represents the first model of the oxidative stress response for a medically relevant microorganism. Our model has allowed us to: (1) predict the existence of additional forms of the key oxidative stress regulators Cap1 and Hog1; (2) highlight the role of catalase as the main detoxification system under the conditions examined; (3) underline the role of glutathione and thioredoxin systems in the maintenance of cellular redox potential and damage repair, respectively; (4) elucidate the collective time- and dose-dependent responses of oxidative stress response mechanisms under different stress scenarios; and (5) assess the effects of key mutations on the ability of *C*. *albicans* cells to respond to oxidative stress.

## Results

### Model Overview

**Fig 1:**
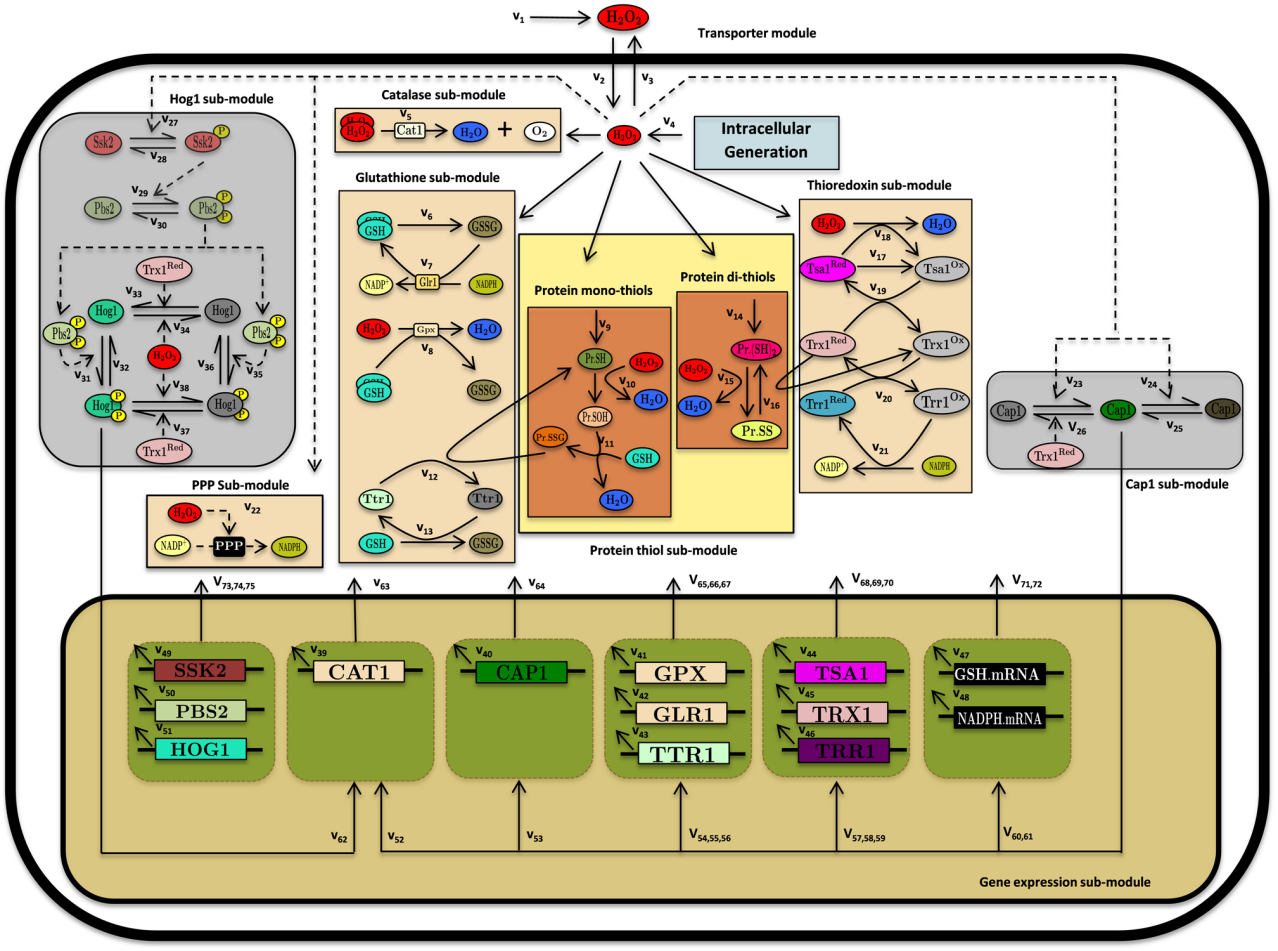
Comprehensive reaction network model of the oxidative stress response in *C*. *albicans*. A description of the modules and sub-modules and the list of components considered in this study are provided in Table 1. The biochemical reactions and system components are presented in S1, S2, S5 and S6 Tables. In addition to the biochemical reactions that are marked in this figure, all components of the model are also assumed to undergo a first order decay. See *Model Construction* for further details.

To understand and predict the dynamics of H_2_O_2_ stress responses in *C*. *albicans* we have developed a quantitative mathematical model that integrates reactions describing catalase-mediated H_2_O_2_ detoxification, the glutathione and thioredoxin systems, NADPH production via the pentose phosphate pathway, the repair of protein damage, and the Cap1 and Hog1 signalling pathways that govern the induction of these systems in response to oxidative stress (Fig 1). The model consists of 120 biochemical reactions (S1 and S2 Tables) that incorporate 45 chemical and biochemical species (S5 and S6 Tables) within two main compartments (the extracellular and intracellular spaces). Fig 1 depicts 75 of these biochemical reactions: the remaining reactions, which describe the decay rates for each of the 45-biochemical species, are included in S1 and S2 Tables.

The 75 biochemical reactions are assigned to four major modules: (i) transporter module; (ii) antioxidant module; (iii) protein thiol module; and (iv) signalling and gene expression module (Table 1). The transporter module describes the kinetics of H_2_O_2_ diffusion through the *C*. *albicans* plasma membrane. Here, exogenously added H_2_O_2_ is presumed to enter the cell via limited diffusion [25,26,27] where it is degraded by catalase. The model also includes the chemical degradation of H_2_O_2_ (S1 Fig). In addition, *C*. *albicans* cells are presumed to generate basal levels of endogenous H_2_O_2_, because even in the absence of exogenously added H_2_O_2_, the deletion of *CAT1* leads to the accumulation of intracellular ROS.

**Table 1:** Cellular modules/sub-modules of the comprehensive reaction network model and their components.

Module	Sub-module	Module components	Description
Transporter module	-	H_2_O_2_^Ex^, H_2_O_2_^In^,	Describes the dynamics of peroxide resulting through the limited diffusion of H_2_O_2_ across the plasma membrane.
Antioxidant module	Catalase	Cat1	Characterises the central H_2_O_2_ detoxification pathway by the enzyme catalase.
	Glutathione	GSH, GSSG, Gpx, Glr1, Ttr1^Red^, Ttr1^Ox^	Describes the roles of the glutathione pathway in maintaining the cellular redox potential, H_2_O_2_ detoxification, and protein mono-thiol repair during oxidative stress.
	Thioredoxin	Tsa1^Red^, Tsa1^Ox^, Trx1^Red^, Trx1^Ox^, Trr1^Red^, Trr1^Ox^	Illustrates the functions of the thioredoxin pathway in H_2_O_2_ elimination and protein di-thiol repair during oxidative stress.
	Pentose phosphate pathway (PPP)	NADPH, NADP	Accounts for the maintenance of the NADPH pool under non-stress and stress conditions
Protein-thiol module	Protein mono & di-thiol	Pr.SH, Pr.SOH, Pr.SSG, Pr.(SH)_2_, Pr.SS	Follows the damage and repair of protein mono-thiols during oxidative stress.
Signalling and gene expression module	Cap1 pathway	Cap1^N^, Cap1^A^, Cap1^I^,	Encapsulates the dynamics of activation of this AP-1-like transcription factors during the oxidative stress response.
	Hog1 pathway	Ssk2, Ssk2.P, Pbs2, Pbs2.PP, Hog1^N^, Hog1^I^, Hog1^N^.PP, Hog1^I^.PP	Tracks the dynamics of this mitogen activated protein kinase pathway during the oxidative stress response.
	Gene expression	*CAT1*, *CAP1*, *GPX*, *GLR1*, *TTR1*, *TSA1*, *TRR1*, *TRX1*, *GSH*, *NADPH*, *SSK2*, *PBS1*, *HOG1*	Accounts for antioxidant gene regulation under normal and oxidative stress a condition.

The antioxidant module tracks the roles of catalase, glutathione and thioredoxin system components in H_2_O_2_ elimination and damage repair. This module is comprised of four sub-modules that describe the activities of these three antioxidant systems, plus their source of NADPH, the pentose phosphate pathway (see *Antioxidant Module* in Materials and Methods).

The catalase sub-module describes the energetically favourable degradation of H_2_O_2_ to water and oxygen by catalase (Cat1). This sub-module has been assigned the major role in H_2_O_2_ elimination because our data show that the inactivation of the single *CAT1* locus in *C*. *albicans* significantly attenuates the rate of decay of extracellular H_2_O_2_ (see *Section B*). In contrast, the inactivation of Glr1 or Trx1, representing the glutathione and thioredoxin systems, did not affect the degradation rates of extracellular H_2_O_2_ under the experimental conditions examined (mid-exponential growth of *C*. *albicans* cells on rich medium [28]). This does not detract from the important roles of the glutathione and thioredoxin systems in the maintenance of redox homeostasis and the repair of oxidative damage under these conditions.

The glutathione (GSH) sub-module describes the role of this abundant tripeptide in the oxidative stress response (see *Glutathione Sub-Module* in Materials and Methods). Yeast and mammalian cells are thought to contain low levels of glutathione disulphide (GSSG) under normal circumstances [29,30,31]. However, under our experimental conditions, we found the ratio of GSH:GSSG to be approximately 2:1 in *C*. *albicans* cells, the total concentration of cellular glutathione being about 9.67 mM. During oxidative stress, the elevated intracellular levels of H_2_O_2_ are reduced by glutathione peroxidase with the concomitant production of GSSG from GSH. Three *C*. *albicans* genes encode glutathione peroxidases (*GPX31-33*), *GPX31* playing the major role in peroxide resistance [32]. Therefore, a single glutathione peroxidase entity is considered in the model, which is represented as Gpx. Oxidized GSSG is then recycled to GSH in an NADPH-dependent manner via glutathione reductase (Glr1) [29]. GSH synthesis is dependent on glutathione biosynthetic genes such as *GCS1*, which like the *GLR1* gene, are induced in response to oxidative stress in a Cap1-dependent fashion [20] (see *Signalling and Gene Expression Module* in Material and Methods).

The thioredoxin sub-module includes the roles of thioredoxin in H_2_O_2_ detoxification and the repair of damaged proteins. Thioredoxin is a highly conserved family of low molecular weight thiol-disulphide oxidoreductases. Of the two thioredoxin genes in *C*. *albicans* (*TRX1*, *TRX2*), only *TRX1* appears to play a major roles in regulating oxidative stress responses and cellular morphogenesis [19,21]. Therefore, the model only includes Trx1, focussing on its role in oxidative stress. (Morphogenesis is not included in the model.) The model describes the direct oxidation of the peroxiredoxin Tsa1 by peroxide following H_2_O_2_ exposure. The resulting oxidized Tsa1 (Tsa1^Ox^) is subsequently reduced by thioredoxin (Trx1^Red^), generating oxidised Trx1 (Trx1^Ox^). Trx1^Ox^ is then recycled back to its native form by the action of thioredoxin reductase (Trr1), which uses NADPH as the reductant. Trx1 also contributes to the repair of oxidised proteins through the formation of intermolecular disulphide bonds [24,33,34,35].

The pentose phosphate pathway (PPP) sub-module portrays the increase in NADPH production that occurs following exposure to oxidative stress to support H_2_O_2_ detoxification by the glutathione and thioredoxin systems. In *S*. *cerevisiae* NADPH production is increased via two mechanisms. First, a rapid increase in NADPH production is mediated by the redirection of metabolic flux through the oxidative branch of the PPP [36]. This is followed by a second increase that occurs through the Yap1-dependent induction of PPP gene expression [37,38,39]. In *C*. *albicans*, two PPP genes (*ZWF1*, *TAL1*) are up-regulated in Cap1-dependent fashion upon exposure to oxidative stress [40]. The PPP sub-module in our model does not detail the pathway in full, but describes these two phases in an abstract fashion, linking PPP gene expression to the amount of activated Cap1 in *C*. *albicans* cells (Fig 1, S1, S2 and S5 Tables: see *PPP Sub-Module* in Material and Methods).

The protein thiol module accounts for the redox reactions of protein mono-thiols and di-thiols during oxidative stress (Fig 1, S1, S2 and S5 Tables: see *Protein Thiol Module* in Materials and Methods). The mono-thiols describe the repair of protein sulfenic acid residues via S-glutathionylation, which is dependent on glutaredoxin (Ttr1), glutathione and NADPH [24,41]. The di-thiols describe the repair of protein disulphides, which are recycled back to their native state by the thioredoxin system [24,33,34,35].

The signalling and gene expression module follows the activation of the Cap1 and Hog1 signalling pathways, and the transcriptional regulation of antioxidant genes under normal and oxidative stress conditions. These are divided into Cap1, Hog1 and gene expression sub-modules.

The Cap1 sub-module describes the reversible, dose-dependent activation of this key regulator in response to oxidative stress. Under normal conditions, this AP-1-like transcription factor exists in its reduced inactive state in *C*. *albicans*, becoming oxidised and activated following exposure to oxidative stress [42,43]. Once intracellular redox homeostasis is restored, Cap1 is presumed to return to its inactive state. In our model we have hypothesised the existence of a third, inactive form of Cap1 for two main reasons. First, a simple two-state theory of Cap1 activation was not sufficient to account for the experimentally observed dose-response curves describing the impact of different H_2_O_2_ concentrations on the activation of Cap1 gene targets. The inclusion of a third inactive form of Cap1, which is theoretically generated reversibly at high doses of oxidative stress, was sufficient to overcome this deficiency (see below). Second, the Cap1 orthologue in *S*. *cerevisiae* (Yap1) is capable of forming distinct oxidised states [44]. Although there has been significant evolutionary rewiring of some regulatory pathways in *C*. *albicans* relative to *S*. *cerevisiae* [18,19,20,7,42], the comparison of Cap1 and Yap1 is apt because they act in analogous manners and mediate analogous responses in these yeasts [18,19,20,42,45]. Also, Cap1 appears to subject to redox regulation in an analogous fashion to Yap1 yeasts [42].

To aptly represent the role of the Hog1 MAPK system during oxidative stress in *C*. *albicans*, we have adopted the well-established description of the Hog1 MAPK model during osmotic stress adaptation in *S*. *cerevisiae* [46,47]. Much of the upstream regulation of Hog1 pathway in *C*. *albicans* during oxidative stress remains obscure. Therefore the model focusses on the MAPK module, and upstream phosphorelay signalling is not included explicitly. A single MAP kinase kinase kinase (Ssk2) [48] and MAP kinase kinase (Pbs2) is responsible for the oxidative stress-mediated activation of Hog1 in *C*. *albicans*. Therefore, similar to the approach of Schaber *et al* [47], the model abstractly describes Ssk2 activation by oxidative stress, its activation of Pbs2, and the subsequent phosphorylation and activation of Hog1. Previous models of the Hog1 MAPK pathway, which addressed its activation in response to osmotic stress, describe three main states for Hog1, namely the native inactive unphosphorylated form, a singly phosphorylated inactive form, and the doubly phosphorylated active form [49,50]. Of these, we only consider the unphosphorylated and doubly phosphorylated forms of Hog1 in order to reduce the complexity of our model. Also, our Hog1 sub-module, which addresses its activation in response to oxidative stress, includes two additional states for Hog1: modified forms of the unphosphorylated and doubly phosphorylated forms of this MAPK. These modified forms of Hog1 are presumed to be generated in response to oxidative stress in a dose-dependent manner, and are presumed to be inactive. The inclusion of these additional forms in a generalised scheme of Hog1 regulation was designed to address the following experimental observations in an unbiased fashion. First, redox-sensitive thiols in Sty1, the *S*. *pombe* orthologue of Hog1, become oxidized following exposure to oxidative stress [51]. Second, *C*. *albicans* Hog1 becomes phosphorylated in response to both oxidative and osmotic stresses and contributes significantly to cellular adaptation to these stresses [14,15]. Third, while contributing significantly to the activation of osmotic stress genes during osmotic stress, Hog1 makes a relatively minor contribution to the induction of oxidative stress genes when subjected to oxidative stress and it appears to act mainly at a post-transcriptional level [19,46]. Therefore, although Hog1 is phosphorylated in response to both oxidative and osmotic stresses, Hog1 contributes differentially to gene activation during adaptation to these stresses (Fig 1, S1, S2 and S5 Tables: see *Hog1 Sub-Module* in Material and Methods).

The activation of Hog1 and Cap1 signalling in response to oxidative stress leads to the induction of key oxidative stress functions, and this is described in the gene expression sub-module. Activated Cap1 induces the transcription of various antioxidant genes that include *CAT1*, *TSA1*, *TRR1*, *TRX1*, *GLR1*, *TTR1* and *CAP1* itself [19,20,40,47]. During peroxide stress, Cap1 also up-regulates specific genes on the pentose phosphate pathway (*ZWF1*, *TAL1*) and the glutathione biosynthetic pathway (*GCS1*) [40]. Moreover, Hog1 activation contributes to the induction of *CAT1* [19]. Hence, in this sub-module these genes are presumed to be transcribed and their mRNAs translated at basal rates under normal conditions, and then induced in a Cap1- and Hog1-dependent fashion in response to oxidative stress. These Cap1 and Hog1 mediated increases in gene expression are modelled using the Hill function, and the resultant increases in protein levels act as inputs for the main antioxidant and protein repair modules. Meanwhile, the rates of Ssk2, Pbs2 and Hog1 production are presumed to be constant during oxidative stress [19].

These various modules and sub-modules are combined into our oxidative stress model such that they track key changes in key cellular redox components, follow changes in the intracellular and extracellular concentrations of H_2_O_2_, and describe the dynamics of Cap1 and Hog1 activation. The model also permits the quantification of oxidative stress-induced changes in cellular redox potential and protein damage. Further details of the model are described in the *Materials and Methods* as well as in the *Supplementary Information* (S1–S6 Tables).

### Contributions of the three main antioxidant systems to H_2_O_2_ detoxification

**Fig 2:**
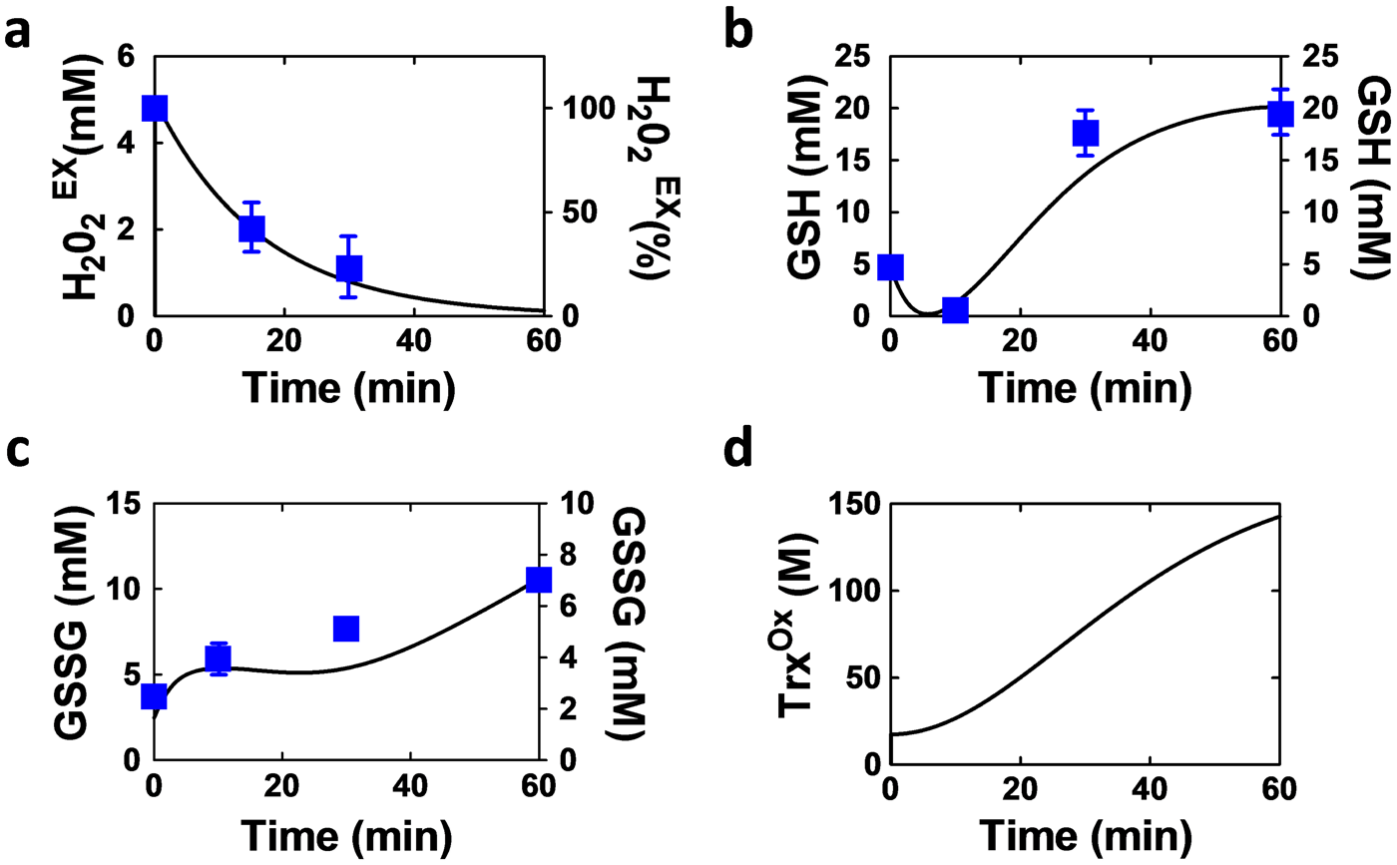
Temporal changes in the levels of stressor, glutathione, glutathione disulphide and thioredoxin following exposure to 5 mM H_2_O_2_. Model simulations (black solid lines) are compared with the corresponding measurements based on three independent experiments (blue boxes). **(a)** Extracellular hydrogen peroxide (H_2_O_2_^Ex^): left hand Y-axis, simulated levels (mM); right hand Y-axis, experimental measurements (percent of initial value). **(b)** Glutathione (GSH) left hand Y-axis, simulated levels (mM); right hand Y-axis, experimental measurements (mM). **(c)** Glutathione disulphide (GSSG) left hand Y-axis, simulated levels (mM); right hand Y-axis, experimental measurements (mM). **(d)** Simulated levels of the oxidised form of thioredoxin (Trx1^Ox^). Experimental errors represent standard deviations from at least three measurements.

During model construction, we tested its ability to reproduce the experimentally measured dynamics of the key components of the catalase and glutathione systems. We found that, following the addition of 5 mM H_2_O_2_ to exponentially growing wild type *C*. *albicans* cells, extracellular H_2_O_2_ levels decreased rapidly, being removed from the growth medium within about 60 minutes (Fig 2a). Meanwhile, the levels of intracellular GSSG increased slowly over this period, and GSH synthesis increased after about 20 minutes (Fig 2b and 2c) [52]. Basal *CAT1* mRNA levels have been reported to be low in the absence of oxidative stress [19]. However, catalase assays [48] and proteomic analyses performed on wild type *C*. *albicans* cells under these growth conditions (Rob Beynon, personal communication) have revealed that high levels of catalase are produced under basal conditions (approximately 1.5 x 10^5^ molecules per cell). Therefore, high basal levels of Cat1 were included in the model, and parameters of the various modules were manually tuned to fit these and other published data (see S2 and S6 Tables). Simulation results of the model displayed good agreement with the experimental data. It is evident from the Fig 2 that the dynamics of the extracellular H_2_O_2_, reduced and oxidised forms of GSH are well tracked by the model. Model simulations suggested an increase in the levels of oxidized Trx1 after exposure to 5 mM H_2_O_2_ (Fig 2d).

**Fig 3:**
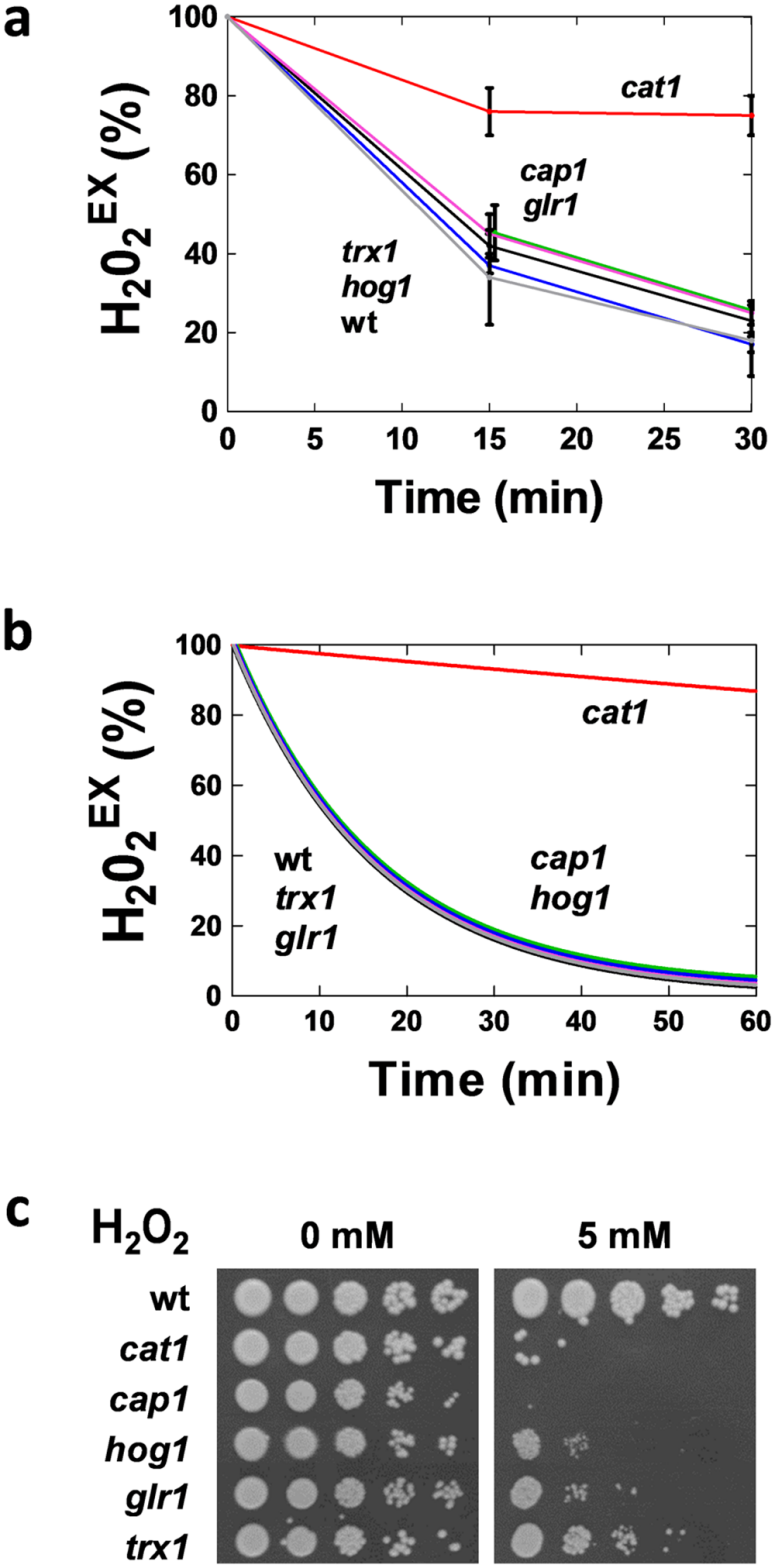
Contributions of the three antioxidant systems to H_2_O_2_ detoxification and resistance. **(a)** Experimental measurements of hydrogen peroxide levels in the medium (H_2_O_2_^Ex^) of mid-exponential *C*. *albicans* cultures following exposure to 5 mM H_2_O_2_, relative to this starting H_2_O_2_^Ex^ concentration (%): wt, *C*. *albicans* wild type (CA372); *cap1* (JC842); *hog1* (JC45); *cat1* (CA1864); *trx1* (JC677); *glr1* (*glr1*Δ/ *glr1*Δ) (S7 Table). **(b)** Model simulations of H_2_O_2_^Ex^ levels following addition of 5 mM H_2_O_2_ to *C*. *albicans* cultures. **(c)** Growth of serial ten-fold dilutions of *C*. *albicans* wild type, *cat1*, *cap1*, *hog1*, *glr1* and *trx1* cells on YPD plates containing H_2_O_2_ after 48 h at 30°C.

We tested the impact of the three main antioxidant systems on the dynamics of ROS clearance, by measuring extracellular H_2_O_2_ levels over time following the addition of 5 mM peroxide to exponentially growing *C*. *albicans* mutants with well-defined deletions in key components of these systems (Fig 3a). The inactivation of Glr1 or Trx1 did not perturb the rapid clearance of H_2_O_2_ from the growth medium, indicating that the glutathione and thioredoxin systems do not play major roles in H_2_O_2_ clearance under these conditions. However, the loss of Cat1 caused a dramatic reduction in the rate of clearance of H_2_O_2_ from the growth medium. This highlights the key role of catalase in peroxide detoxification in the oxidative stress response network. Interestingly, the rates of H_2_O_2_ clearance were normal for *cap1* and *hog1* cells, in which the key oxidative stress signalling pathways have been blocked (Fig 3a). This was consistent with the idea that H_2_O_2_ clearance is primarily dependent on the high basal levels of catalase that exist in *C*. *albicans* cells under the growth conditions examined. These experimental observations were used to parameterise the model, yielding simulation results that were in good agreement with the experimental data. The predicted dynamics of H_2_O_2_ clearance following the conceptual inactivation of specific model components were consistent with the experimentally determined dynamics for the corresponding deletion mutants (Fig 3b).

The relatively minor roles for Cap1 and Hog1 signalling and the glutathione and thioredoxin systems in H_2_O_2_ clearance immediately following exposure to oxidative stress do not detract from their key roles in the restoration of redox homeostasis and the repair of oxidative damage during oxidative stress adaptation. Indeed, we found that like *cat1* cells, the *glr1*, *trx1*, *cap1* and *hog1* mutants displayed significantly reduced growth on plates containing 5 mM H_2_O_2_ compared to the wild type control (Fig 3c). This recapitulates previous reports describing the oxidative stress sensitivity of these *C*. *albicans* strains [13,14,15,53]. Thus the roles of Cap1, Hog1, Glr1 and Trx1 in oxidative stress adaptation are captured in the model (S1 and S2 Tables: see *Model Construction* in Materials and Methods).

### A third, inactive form of Cap1 can account for oxidative stress dose response curves

Enjalbert *et al*. have reported dose response curves for oxidative stress response genes in *C*. *albicans* [54]. These dose response curves were based on the levels of green fluorescence expressed by *C*. *albicans* cells transformed with specific promoter-GFP fusions, two hours after exposure to a given concentration of H_2_O_2_. The *CAT1*_*pr*_ *-GFP* (previously called *CTA1)*, *TTR1*_*pr*_ *-GFP* (also called *GRX2*) and *TRX1*_*pr*_ *-GFP* fusions showed near linear increases in the levels of green fluorescence in the range of 0–2 mM H_2_O_2_. However, the expression of these GFP fusions declined at H_2_O_2_ concentrations above this range, reaching a minimal value for the highest dose tested (30 mM H_2_O_2_) [54]. The basis for this unexpected behaviour has remained unclear. We addressed this conundrum using a combination of experimentation and modelling.

**Fig 4:**
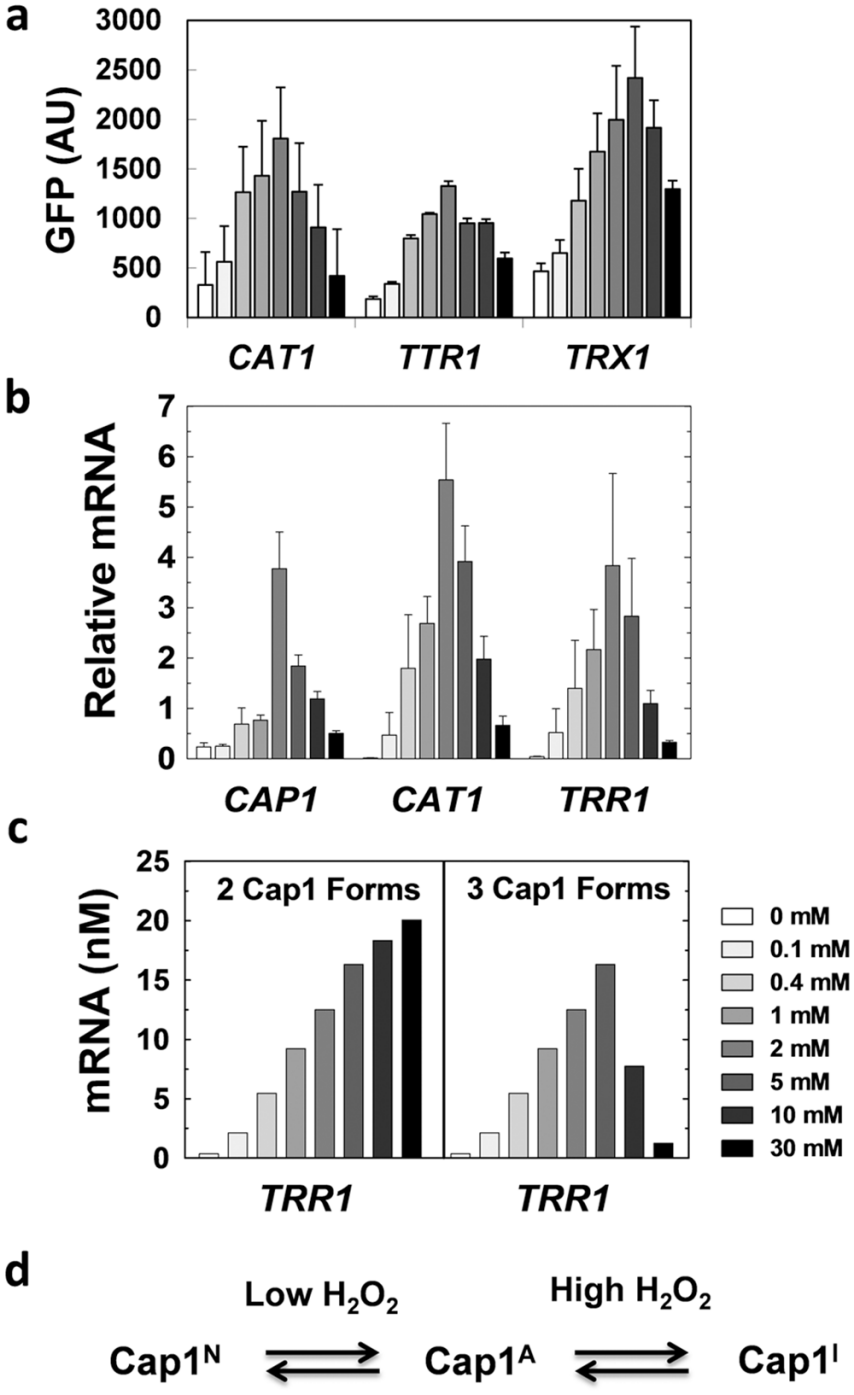
Dose-dependent response of Cap1-dependent genes following exposure to different H_2_O_2_ concentrations. **(a)** Data from Enjalbert *et al*. [54] showing GFP expression levels from *CAT1-*, *TTR1*-, and *TRX1-GFP* reporters in *C*. *albicans* cells exposed to a range of H_2_O_2_ concentrations (grey scale, bottom right). GFP intensities are expressed in absorbance units. **(b)** Data from this study showing the levels of *CAP1*, *CAT1* and *TTR1* mRNAs in *C*. *albicans* cells exposed the same range of H_2_O_2_ concentrations. Relative mRNA levels were measured by qRT-PCR relative to the internal *ACT1* mRNA control. We show data of three independent experiments and the corresponding SD (t-test). **(c)** Simulation results for *TRR1* mRNA levels (nM) after exposure to different H_2_O_2_ concentrations, obtained using oxidative stress models that lack (2 Cap1 Forms) or include the third conceptual form of Cap1 (3 Cap1 Forms). **(d)** Proposed model of Cap1 regulation in *C*. *albicans*.

The data generated by Enjalbert *et al*. were obtained using GFP, which is a relatively stable protein [54]. Hence it was conceivable that these dose response curves might not accurately reflect the actual behaviours of the wild type genes. Therefore, we tested experimentally whether a wild type oxidative stress transcript displays an analogous dose response curve to the corresponding GFP fusion. The levels of the wild type *TRR1* transcript were measured in *C*. *albicans* cells, 10 minutes after exposure to a range of H_2_O_2_ concentrations. The resulting dose response curve was remarkably similar to that obtained previously for a *TTR1*_*pr*_ *-GFP* fusion (Fig 4a). Oxidative stress gene induction displayed an optimum level of induction at an H_2_O_2_ concentration of about 5 mM (Fig 4b).

Our attempts to recapitulate this dose response behaviour by modelling failed when the models contained the two forms of Cap1 described in the literature (Fig 4c): reduced inactive Cap1, and oxidized active Cap1 [42,55]. Therefore, we considered the analogy with *S*. *cerevisiae* Yap1, for which several different oxidized forms of the protein have been observed [44]. We hypothesised that the attenuated induction of oxidative stress genes at high doses of stress could be explained by the generation of a third, inactive form of Cap1 at these high doses of H_2_O_2_ (Fig 4d). Therefore, we tested the impact of introducing a third, inactive form of Cap1 into the Cap1 sub-module. Model simulations were performed for the range of 0–30 mM H_2_O_2_, and predicted the *TRR1* mRNA levels 10 minutes after peroxide exposure (Fig 4c). These simulation results display similar patterns to the experimentally determined dose response curve for the wild type *TRR1* mRNA (Fig 4a). Consequently, three main forms of Cap1 exist in our model of oxidative stress adaptation in *C*. *albicans*: (i) an inactive reduced form that is present under basal conditions; (ii) an active oxidised form that is generated in response to oxidative stress; and (iii) a third, inactive form of Cap1 that is generated at high doses of oxidative stress (Fig 4d).

**Fig 5:**
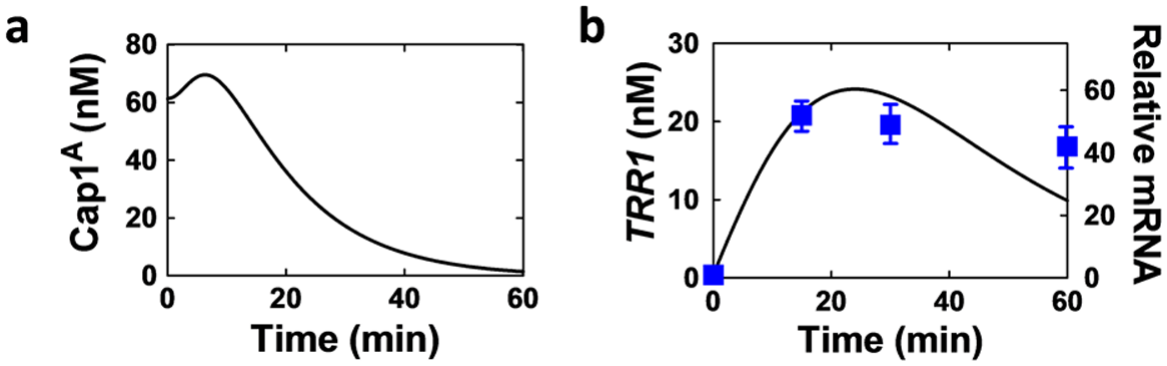
Temporal changes in the levels of (a) active Cap1 (Cap1^A^) and (b) *TRR1* mRNA levels following exposure to 5 mM H_2_O_2_. Model simulations are represented by black solid lines (left hand Y-axes), and experimental measurements by blue boxes (three independent experiments; right hand Y-axis). Relative *TRR1* mRNA levels were measured relative to the internal *ACT1* mRNA control. Standard deviation was calculated and is shown in the figure.

We simulated the dynamics of Cap1 activation (i.e. the levels of Cap1^A^) following exposure to 5 mM H_2_O_2_ (Fig 5a). These simulation results are entirely consistent with the time course of Cap1 activation and inactivation reported by Patterson *et al* [43]. In addition, we simulated the dynamical changes in mRNA levels for key Cap1 target genes in *C*. *albicans* cells following exposure to 5 mM H_2_O_2_. These simulation results accurately reflected our measurements for such mRNAs. Fig 5b shows both simulated and measured *TRR1* mRNA levels under these experimental conditions (Fig 5b). These data reinforce the validity of our Cap1 representations in the oxidative stress response model.

### Oxidative stress dose response curves are affected by catalase inactivation

**Fig 6:**
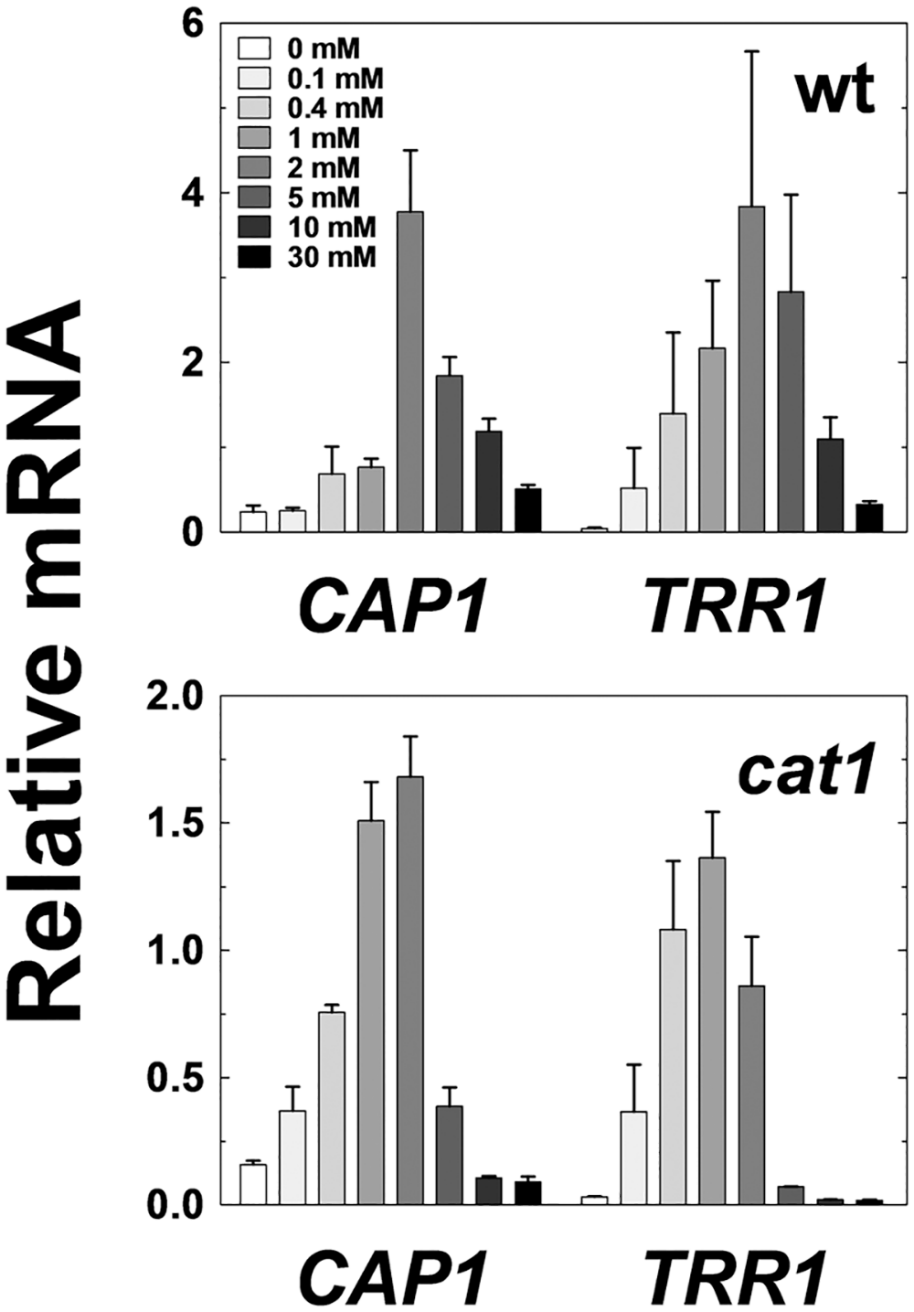
Catalase (Cat1) inactivation shifts the dose response curve to lower H_2_O_2_ concentrations. Using qRT-PCR, relative *CAP1* and *TRR1* mRNA levels were measured relative to the internal *ACT1* mRNA control in *C*. *albicans* cells exposed the same range of H_2_O_2_ concentrations examined in Fig 4 (grey scale, top left): upper panel, wt (wild type, CA372); lower panel, *cat1* (CA1864) (S7 Table). The data is representative of three independent experiments and the standard deviation was calculated.

Having constructed an integrated model of the oxidative stress response in *C*. *albicans*, we then used this model to explore oxidative stress adaptation in this pathogen. We had shown that catalase is essential for the rapid clearance of H_2_O_2_ from the growth medium by *C*. *albicans* (Fig 3a). Also, *C*. *albicans cat1* cells are sensitive to oxidative stress (Fig 3c) [53]. Therefore, we reasoned that the oxidative stress dose response curve for a *cat1* mutant should be qualitatively different from that observed for wild type cells. Specifically, the model predicted that *C*. *albicans* cells lacking catalase would experience a higher oxidative stress than wild type cells at an equivalent dose of H_2_O_2_. Consequently, the third, inactive form of Cap1 is generated at lower concentrations of oxidative stress, and as a result, the dose response curve for *cat1* cells reaches a maximum at a lower H_2_O_2_ concentration than for wild type cells. To test this used qPCR to measure the levels of the *TRR1* and *CAT1* transcripts under equivalent experimental conditions (Fig 6). The simulations confirmed our expectations: the predicted maxima for the *TRR1* dose response curves in *cat1* and wild type cells were 0 and 5 mM H_2_O_2_, respectively. Our experimental observations confirmed this downward shift in the oxidative stress dose response curve for *cat1* cells towards lower H_2_O_2_ concentrations, although the actual shift was not as dramatic as that predicted by the model. As expected, no *CAT1* transcript was predicted or observed in the *cat1* deletion mutant (not shown). The simulations and experimental data show reasonable agreement. Therefore, the model successfully predicted this previously untested behaviour of the oxidative stress system in *C*. *albicans*.

### Redox Potential (ΔE) as a qualitative proxy for oxidative stress sensitivity

The model tracks the dynamic behaviours of oxidative stress components, but does not link these directly to cell death. Exposure to high levels of H_2_O_2_ is known to trigger programmed cell death and necrotic death, and the frequency of killing is influenced by Ras-cAMP signalling [56,57]. However, the mechanistic basis for oxidative stress killing in *C*. *albicans* cells has not been established. The GSSG/GSH redox potential is often used as a measure of disturbances in redox metabolism, and increases in redox potential (ΔE) above about -180 have been associated with oxidant-driven apoptosis [58,59]. Therefore, we considered the use of the redox potential (ΔE) as a qualitative indicator of oxidative stress killing, whereby significant increases in ΔE above -180 are indicative of increased frequencies of cell death.

**Fig 7:**
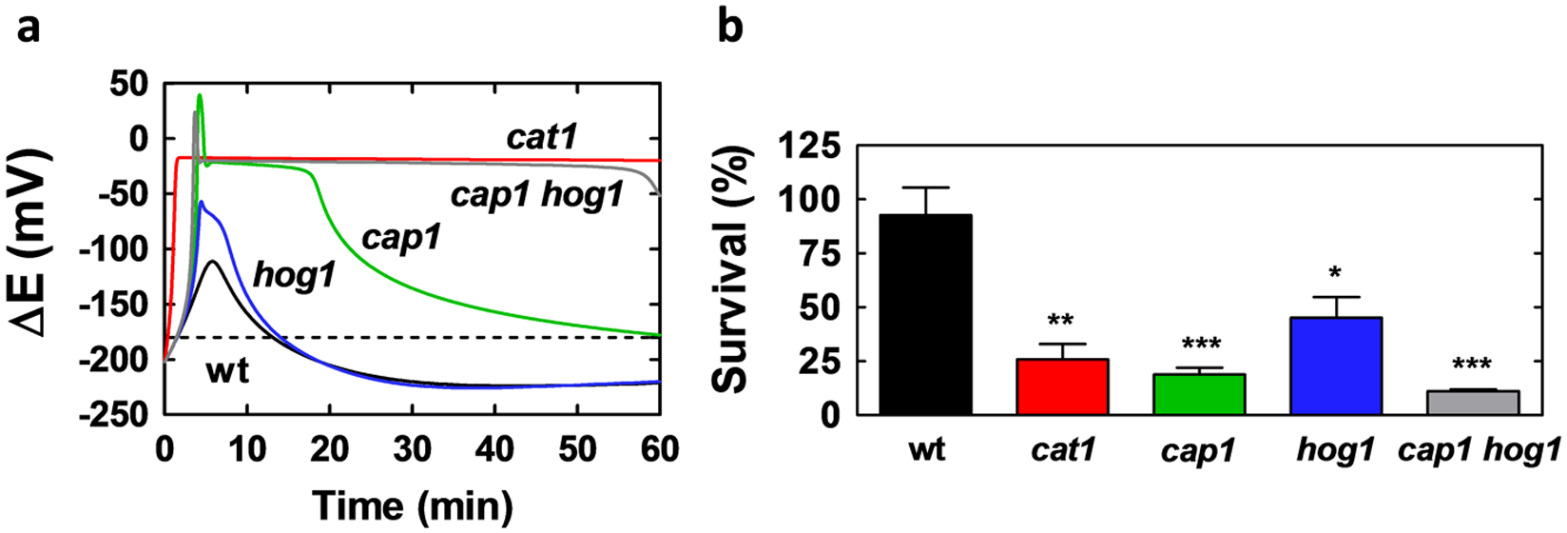
Perturbation of Redox Potential (ΔE) is a reasonable proxy for oxidative stress sensitivity. **(a)** Simulated changes in ΔE in *C*. *albicans* cells following exposure to 5 mM H_2_O_2_: wt, wild type (CA372); *cap1* (JC842); *hog1* (JC45); *cat1* (CA1864) and *cap1 hog1* (JC118) (S7 Table). The dotted line represents -180 mV, above which cells are more likely to enter oxidant-driven cell death pathways [58,59]. **(b)** Experimental determination of percentage survival following exposure of the *C*. *albicans* strains to 5 mM H_2_O_2_: *, P<0.05; **, P<0.01; ***, P<0.001, using an Unpaired t-test.

The dynamical behaviour of the redox potential following exposure to 5 mM H_2_O_2_ was simulated for wild type cells and for *C*. *albicans* mutants lacking key components of the oxidative stress response: Cap1, Hog1 and Cat1. The redox potential ΔE in wild type cells was predicted to increase transiently above -180 (Fig 7a). However, the change in ΔE was much greater for *cap1*, *hog1* and *cat1* cells. This correlated with the increased sensitivity of these mutants to 5 mM H_2_O_2_ (Fig 7b). Furthermore, the model predicted that the perturbation in redox potential would be more significant for *cat1* and *cap1* cells than for *hog1* cells, essentially because of the major roles assigned to catalase and Cap1 in oxidative stress adaptation. This was entirely consistent with the experimental observation that *cat1* and *cap1* cells are more sensitive to killing by oxidative stress than *hog1* cells (Fig 7b). Therefore, significant perturbation of the redox potential ΔE over the *circa* 10 minute period following the imposition of the stress provides a qualitative indication of increased oxidative stress sensitivity. However, it does not provide a quantitative measure of this sensitivity.

### Transient molecular memory following sequential stresses

Cellular memory is an inherent property of biological systems [45,60,61]. With respect to oxidative stress, *C*. *albicans* cells that are pre-treated with a low dose of oxidative stress are known to be protected from the deleterious effects of a subsequent, high dose of the stress [11,62]. This adaptation is thought to be mediated by the induction of key oxidative stress functions in response to the first stress, which then provide some degree of protection against the second stress. Therefore, we tested whether our model was capable of predicting this adaptation to oxidative stress, and if so, whether the model predicted the duration of this protective effect (i.e. whether this molecular memory is transient).

**Fig 8:**
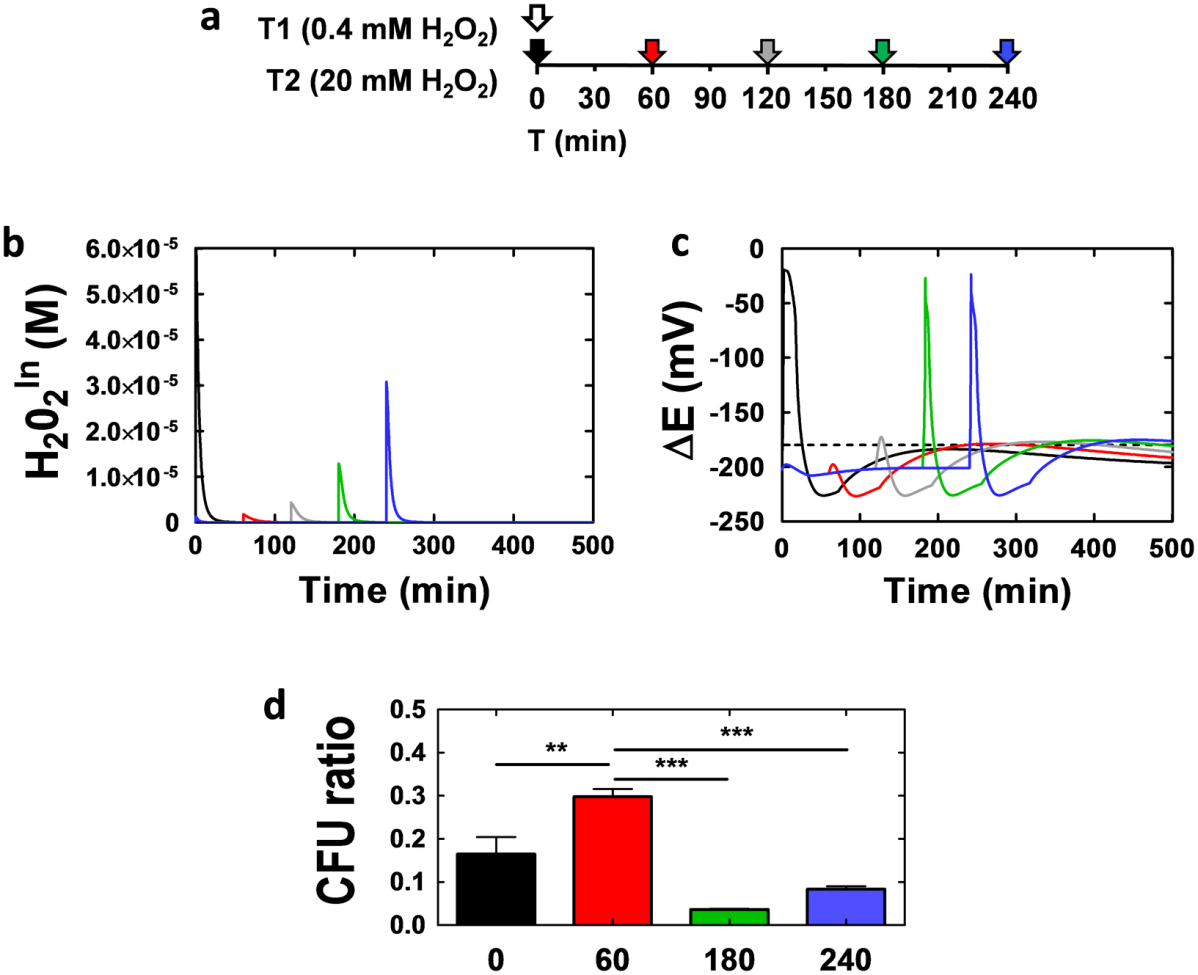
The model accurately predicts the temporal protection provided by oxidative stress adaptation. **(a)** Representation of the timing of the sequential stresses applied in the experiment. The white arrow represents the initial 0.4mM mM H_2_O_2_ stress (T1), whilst the colored arrows represent the addition of the second 20 mM H_2_O_2_ stress (T2) at the following times after the first stress: 0 min (black arrow); 60 min (red arrow); 120 min (grey arrow); 180 min (green arrow); 240 min (blue arrow). **(b)** The predicted dynamics of intracellular H_2_O_2_ levels (H_2_O_2_^In^), and **(c)** redox potential (ΔE) after imposition of the second 20 mM H_2_O_2_ stress at: 0 min (black); 60 min (red); 180 min (green); 240 min (blue). **(d)** Experimentally measured survival of *C*. *albicans* cells after exposure to the sequential stresses when T2 was: 0 min (black); 60 min (red); 180 min (green); 240 min (blue). Colony forming units (CFU) for stressed cells were measured relative to the untreated control cells. The data represent mean and standard deviation values from three independent experiments: *, P<0.05; **, P<0.01.

First, we simulated the exposure of *Candida* cells to two sequential oxidative stresses: first cells were exposed conceptually to 0.4 mM H_2_O_2_, and then after a given period (T) these cells were exposed to a second dose of 20 mM H_2_O_2_ (Fig 8a). These simulations highlighted the high levels of intracellular H_2_O_2_ that cells are likely to be exposed to when T = 0, i.e. the 0.4 mM and 20 mM doses of H_2_O_2_ are imposed simultaneously (Fig 8b). However, when *C*. *albicans* cells have had time to adapt to the initial dose of 0.4 mM H_2_O_2_ (T = 60 minutes), the intracellular ROS levels generated by the second 20 mM H_2_O_2_ stress are much lower. Also, the degree of protection against high intracellular ROS then declines as the period between the two stress impositions is increased (Fig 8b). Consequently, the model predicts that that there is a similar protective effect for the redox potential ΔE (Fig 8c). Simultaneous imposition of the two stresses results in a significant perturbation of the redox potential. However, prior exposure to 0.4 mM H_2_O_2_ for 60–120 minutes provides significant protection against the subsequent addition of 20 mM H_2_O_2_. Our modelling predicts that this protection is lost if the period between the two stresses is increased beyond 120 minutes (Fig 8c). Therefore, our modelling successfully predicts that oxidative stress pre-adaptation protects *C*. *albicans* cells against high doses of this stress. Furthermore, it predicts that this protection is transient, lasting only for a few hours.

We tested these predictions experimentally by measuring the effects of altering the time (T) between exposure of *C*. *albicans* cells to a first (0.4 mM) and second (20 mM) H_2_O_2_ upon the viability of these cells (Fig 8d). We focused on those key time points that were predicted by the modelling to display significant differences (0, 60, 180 and 240 min). As expected, a relatively low number of cells survived the imposition of these two stresses simultaneously (T = 0), i.e. most cells were killed. However, about twice as many cells survived exposure to 20 mM H_2_O_2_ when they had been exposed to 0.4 mM one hour beforehand (T = 60). As predicted by the model, this protection was lost if this period was extended to 180 or 240 minutes (Fig 8d). Therefore, our model of oxidative stress adaptation, which was built using data obtained following exposure to a single stress, successfully predicted responses to sequential stresses and the temporary nature of the molecular memory that arises from oxidative stress adaptation.

### Sensitivity analysis

We performed a sensitivity analysis of the model to identify the set of parameters with the greatest influence on the model predictions. The analysis was performed by varying the model parameters over two orders of magnitude and computing the relative error measures of the model predictions with respect to the experimentally measured values of the concentration of extracellular hydrogen peroxide (H_2_O_2_^Ex^) and antioxidant system components (i.e. *GSH*, catalase and *TRR1* mRNA levels), chosen as representative sensitivity measures of the system (for further details see *Sensitivity Analysis* in Material and Methods). This analysis identified the parameters corresponding to H_2_O_2_ permeability and mRNA induction rates to be the most sensitive parameters of the model.

**Fig 9:**
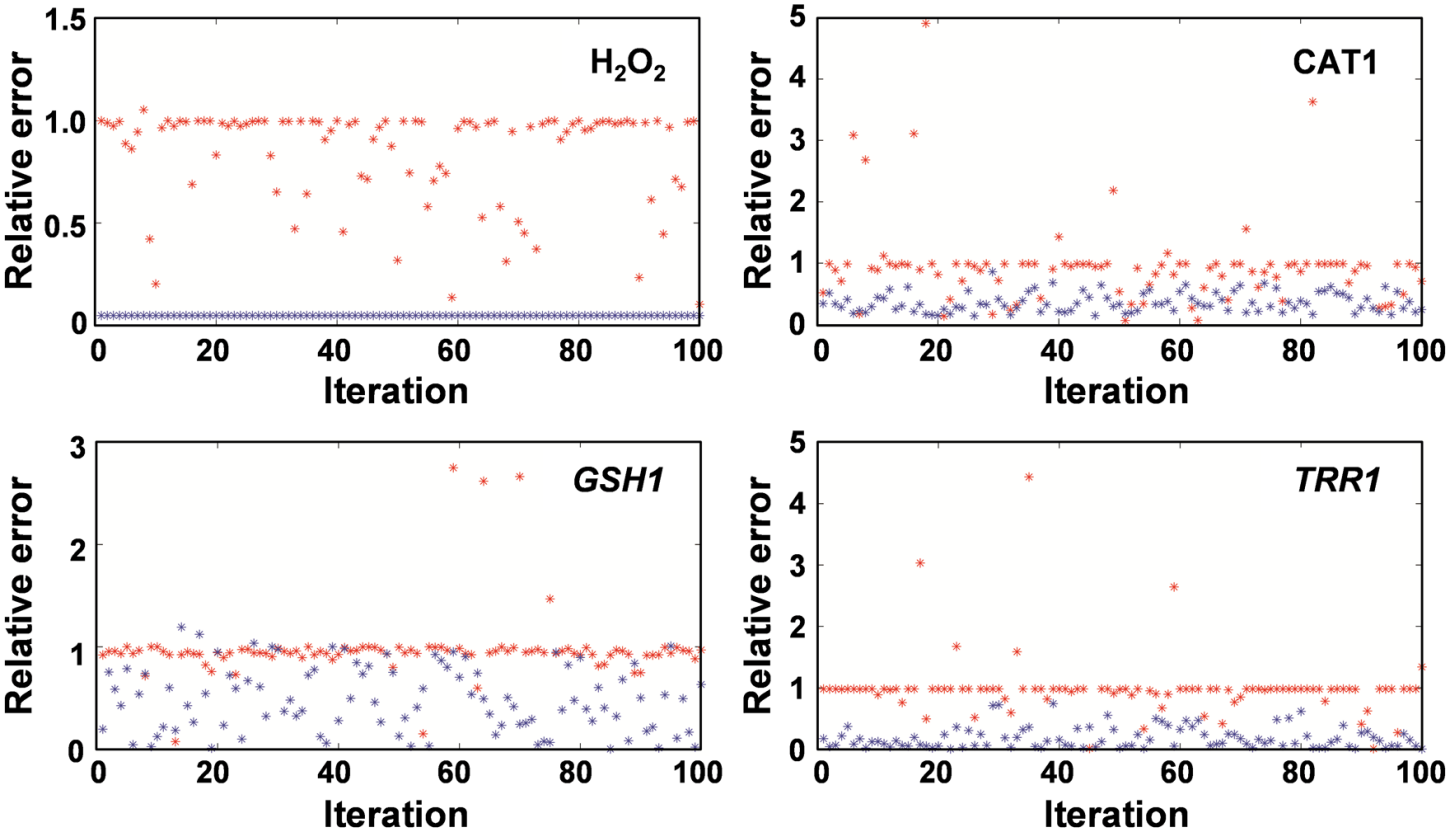
Permeability and mRNA induction rates are the most sensitive parameters of model. Relative error values in catalase (CAT1) at 10 min, *TRR1* mRNA levels at 10 min, H_2_O_2_^Ex^ at 15 min, and GSH at 30 min estimated for 100 different parameter sets. Red points correspond to the case when the set of 73 key reaction rates were varied according a homogenous random distribution between 0.1 and 100 their nominal value, and blue points represents the case when both permeability and mRNA induction rates were fixed to their nominal values, whereas the rest of the parameters were randomly varied as above.

Initially we generated 100 different sets of parameters by varying simultaneously the reaction rates of all antioxidant systems according to a homogeneous random distribution between 0.1 and 10 times their nominal value. Then, for each set of parameters, we calculated the relative error for the four chosen sensitivity measures (H_2_O_2_^Ex^, *CAT1*, *GSH* and *TRR1*), resulting in relative errors very close to 1 for most of the random sets of parameters. This indicates that the simulated values of the model components with the generated random set of parameters decay too rapidly, as verified by visual inspection of the simulations (red dots in Fig 9, S2 and S3 Figs).

We then set out to identify the most sensitive model parameters by pre-selecting groups of parameters corresponding to one of the main oxidative stress response system modules and fixing them to their nominal values, whilst varying the rest of the model parameters in the same manner as before (see *Sensitivity Analysis* in Material and Methods). This analysis identified the permeability rates and the parameters corresponding to the mRNA induction rates as the most sensitive ones, as the relative error was reduced considerably when we fixed these mRNA induction rates (S2 and S3 Figs, blue dots). Importantly, by fixing the permeability rates the error decreases only for the external H_2_O_2_, whereas by fixing the mRNA induction rates, the error decreases in all sensitivity measures but the external H_2_O_2_. This result is consistent with the experimental observation that the clearance of the external H_2_O_2_ signal depends primarily on the basal level of Cat1 (which was fixed in all simulations) and not the induced levels of mRNA (Fig 3). Hence, in Fig 9 we present the results for the case in which we fix simultaneously the permeability rates and the mRNA induction parameters. As expected, now the error decreases considerably for all sensitivity measures.

In summary, the sensitivity analysis identifies the permeability rates of H_2_O_2_ and the mRNA induction parameters upon the oxidative stress signal as the most sensitive parameters of the model.

## Discussion

In this study we have used integrative systems approaches to examine the oxidative stress response in *C*. *albicans*. Our analyses add significantly to previous studies which have modelled oxidative stress responses in other systems. For example, Klipp’s group examined the impact of superoxide dismutase in ROS detoxification, providing a possible explanation for the unexpected increases in lipid peroxidation observed in bacteria and mammalian cells following superoxide dismutase overexpression [63,64]. Ralser and co-workers have used mathematical modelling to examine the role of central carbohydrate metabolism during oxidative stress in *S*. *cerevisiae* [65]. Their metabolic model corroborated experimental observations which indicate that the redirection of carbon flux from glycolysis to the pentose phosphate pathway, which enhances NADPH production, is important to counteract oxidative stress in this yeast [36,65]. Another study has exploited mathematical modelling to describe the differential changes that were observed in the activities of antioxidant enzymes during H_2_O_2_ stress in mammalian cells [66]. In contrast to *S*. *cerevisiae* and *C*. *albicans*, these authors observed no changes in catalase levels in cultured colon (Caco-2) cells following H_2_O_2_ exposure [66]. It has been reported that catalase is present on the *C*. *albicans* cell wall [67]. However, these authors provided no evidence to suggest that this catalase is active, and we did not detect catalase activity in the culture supernatants (not shown). Furthermore, existing models that describe the dynamics of intracellular H_2_O_2_ do not consider the possibility of extracellular catalase activity [24,68,69]. Therefore, our model does not include extracellular catalase.

More recently, Adimora and co-workers have described a kinetic model that examines the transient oxidation of the thiol proteome following oxidative stress in mammalian (Jurkat) cells, and the protection offered by the glutathione and thioredoxin systems [24]. They predicted that the thioredoxin system plays the major role in protecting and repairing protein thiols following oxidative damage. Our work extends these studies by being the first to describe an oxidative stress model that integrates the major signalling cascades, changes in gene expression, the three main antioxidant systems, and their impact on cellular protein thiols and redox homeostasis in a eukaryotic system. Furthermore, it is the first to model oxidative stress adaptation in a major fungal pathogen of humans.

We have found an integrative systems approach to be particularly useful during our examination of the oxidative stress response. This response involves a range of cellular processes that operate collectively to detoxify the stress, repair oxidative damage and restore cellular homeostasis. Furthermore, the relative contributions of these processes change over time. Therefore, it can be challenging to define the impact of one specific process on the overall response using experimental approaches alone. These approaches often provide a relatively focused view of one particular pathway, often examining the phenotypic effects of null mutants at a selected time points. Possible impacts of these null mutations upon other pathways are not generally considered. In contrast, a comprehensive systems-level approach can contribute significantly to our understanding of the system by integrating the inputs from key modules and by predicting the dynamic changes in system behaviour over time.

There are several inherent challenges in the construction of large mathematical models that integrate many cellular processes. Firstly, it can be difficult to establish the boundaries of the individual modules within the model as the cellular processes they describe are often highly interconnected. Secondly, in general it is not possible to include every single component of a particular cellular pathway. Also, this increases model complexity and can result in a model that is intractable. Thirdly, it is challenging to estimate the reaction rates and constants from the limited experimental datasets that are available. Nevertheless, integrative models have contributed significantly to our understanding of yeast adaptive responses [65,49,50,70], and have proven valuable in our studies of the oxidative stress response.

In particular, our integrative systems approach has highlighted the relative significance of the main antioxidant systems and their changing contributions over time following H_2_O_2_ exposure. Previous molecular and cellular studies had demonstrated the critical importance of Cap1 and Hog1 signalling in oxidative stress resistance, and their key roles in inducing oxidative stress genes in *C*. *albicans*, including *CAT1* [11,12,13,14,15]. Furthermore, the experimental dissection of the oxidative stress response in *C*. *albicans* had highlighted the importance of the thioredoxin and glutaredoxin systems in repairing oxidative damage and restoring redox homeostasis [32]. This led to the generally accepted perception that oxidative stress genes are expressed at relatively low basal levels in *C*. *albicans*, and that following ROS exposure, the initial cellular response involves the activation of Cap1 and Hog1 signalling to increase oxidative stress gene expression, leading ultimately to recovery and adaptation [7]. Consequently, our early models assumed constant H_2_O_2_ levels over the *circa* four hour timescales we were examining. These assumptions were quickly challenged as our experimental tests failed to validate our model predictions. We found that *C*. *albicans* expresses relatively high basal levels of catalase in the absence of stress, that this catalase is critical for the rapid detoxification of H_2_O_2_ by *C*. *albicans* cells, and that neither Cap1 nor Hog1 are required for this rapid H_2_O_2_ detoxification (Fig 3). Therefore, catalase plays a critical role in the oxidative stress response immediately after H_2_O_2_ exposure, playing the major role in H_2_O_2_ detoxification and the rapid down-regulation of the input ROS signal over time. Following H_2_O_2_ detoxification, the glutaredoxin and thioredoxin systems play important roles in the restoration of redox homeostasis and the repair of oxidised protein thiols. Thus perturbation of either of these systems, for example by inactivation of Glr1 or Trx1, attenuates oxidative stress resistance (Fig 3c), as reported previously [21,32].

Our modelling has also predicted the existence of new functionally distinct forms of key oxidative stress regulators. Alternative forms of Hog1, including an inactive modified phosphorylated form of Hog1, were incorporated in the model to account for this important observation: there are significant differences in Hog1 transcriptional outputs in response to both oxidative and osmotic stress, but Hog1 is phosphorylated under both conditions [15,19]. The hypothesis that Hog1 can be modified is supported by the precedent that the Sty1 stress activated protein kinase becomes oxidised when *S*. *pombe* cells are exposed to ROS [51]. In addition, our model predicts the existence of a third, inactive form of Cap1 which is formed at relatively high doses of oxidative stress (Fig 4). Recent data indicate that the activation of Pap1 in *S*. *pombe* is inhibited at high doses of H_2_O_2_ via the generation of an oxidised sulphinic form of Tpx1 [32]. This form is relatively resistant to reduction by Trx1, thereby releasing Trx1 to reduce other substrates such as Pap1. Therefore, an alternative two-state Cap1 model is conceivable, in which high doses of H_2_O_2_ block Cap1 oxidation. Nevertheless, the three-state Cap1 model is supported by the observation that alternative forms of *S*. *cerevisiae* Yap1, the orthologue of *C*. *albicans* Cap1, are generated in response to higher doses of oxidative stress [44]. The inclusion of a third, inactive form of Cap1 was sufficient for the model to replicate the experimentally observed dose responses for Cap1 target genes in *C*. *albicans* (Fig 4). The model also correctly predicted that the inactivation of catalase, which is critical for rapid H_2_O_2_ detoxification, causes a downward shift in the maximum for this dose response curve to lower H_2_O_2_ concentrations (Fig 6).

The oxidative stress model was constructed using data generated using single doses of H_2_O_2_. Nevertheless, using the model we were able to predict responses to sequential oxidative stresses, i.e. the protective effects of adaptation to an initial low dose of H_2_O_2_ against a subsequent large dose of the same stressor (Fig 8). Cell survival is not included within the boundaries of the model. Therefore, to estimate oxidative stress protection, we first had to identify a molecular proxy within the model that qualitatively reflects cell survival following H_2_O_2_ exposure. We found that the degree of perturbation of the redox potential (ΔE) was a reasonable proxy for cell survival (Fig 7). Using the model, *C*. *albicans* were then conceptually treated with a low dose of H_2_O_2_ (0.4 mM), and then exposed to a high dose (20 mM H_2_O_2_) at different times thereafter. If there was no period of adaptation between these stresses, cells would be expected to be exposed to large spikes of intracellular ROS and consequently suffer major perturbations to their redox potential (Fig 8). However, adaptation times of 60 to 120 minutes were predicted to provide considerable protection, resulting in minimal redox perturbation. The model also predicted that this protection is transient, being lost after 180 minutes (Fig 8C). Our experimental analyses of adaptive protection confirmed these modelling predictions: treatment of *C*. *albicans* cells with 0.4 mM H_2_O_2_ one hour before exposure to 20 mM H_2_O_2_ provided a significant degree of protection against this acute stress, but this protection was significantly reduced when the adaptation time was extended to 180 minutes and beyond (Fig 8D). Therefore, the model correctly predicted the transient nature of the molecular memory generated by oxidative stress adaptation.

The ability of this model to predict the dynamics of oxidative stress responses in *C*. *albicans* with reasonable accuracy is significant for several reasons. First, the model may be exploited to generate useful insights into the mechanics and dynamics of oxidative stress adaptation under a wide range of scenarios *in vitro*. Second, oxidative stress adaptation is critical for the pathogenicity of *C*. *albicans*, playing key roles in protecting this yeast against phagocytic killing for example. Most investigations into the role of *C*. *albicans* oxidative stress responses as the fungus interacts with macrophages or neutrophils have exploited *C*. *albicans* null mutants that lack key components of the oxidative stress response [32,43]. These represent relatively sophisticated molecular approaches, but they are unable to provide detailed insights into the probable dynamics of the oxidative stress responses in fungal cells as they combat the oxidative insults of attacking phagocytes. Therefore, in addition to providing information about oxidative stress adaptation *in vitro*, mathematical models such as the one presented in this paper may also provide valuable insights into the dynamics of fungus-host interactions. Indeed, this model may be extended to include responses to other physiologically relevant environmental insults. Finally, although *C*. *albicans* is more resistant to stresses than many other yeast species [12], many components of the model are likely to be of some utility in other systems.

## Materials and Methods

### Strains and Growth Conditions

The strains used in this study are listed in S7 Table. *C*. *albicans* cells were grown overnight at 30°C, 200 rpm in YPDT (Tris buffered YPD: 2% w/v glucose, 2% w/v mycological peptone, 1% w/v yeast extract, 100 mM Tris.HCl, pH 7.4: [28]), and then reinoculated into fresh pre-warmed YPDT and regrown to an OD_600_ of 1 at 30°C, 200 rpm. On the day of the experiment, these cells were inoculated in fresh pre-warmed YPDT to an OD_600_ of 0.2, and grown an OD_600_ of 0.8, as described previously [28]. Oxidative stress was then applied by adding hydrogen peroxide (H_2_O_2_) at the specified concentrations.

### Stress sensitivity

The stress sensitivities of *C*. *albicans* strains were assayed in two ways. First, the growth of strains was monitored after serial dilution of overnight cultures, plating these dilutions onto YPD agar containing the stress, and incubating these plates at 30°C for one day [15]. Second, mid-exponential *C*. *albicans* cells were treated with specific concentrations of H_2_O_2_ for given periods, and the percentages of viable cells surviving the stress were determined by measuring CFUs on YPD plates lacking the stress [48].

### Peroxide Assays

*C*. *albicans* wild type (CA372), *cat1* (CA1864), *cap1* (JC842), *hog1* (JC45), *glr1* (*glr1Δ/glr1Δ*) and *trx1* (JC677) cells (S7 Table) were grown to exponential phase in YPDT and exposed to 5 mM H_2_O_2_. The concentrations of H_2_O_2_ in the medium were determined at the specified time points using the QuantiChrom peroxide assay kit (BioAssay Systems; Universal Biologicals, Cambridge, UK) following the manufacturer’s instructions.

### Glutathione Assays

Glutathione (GSH) and glutathione disulphide (GSSG) levels were assayed by LC-MS/MS by adapting previously described procedures [71]. Briefly, mid-exponential *C*. *albicans* cells (above) were harvested at the specified time points, and proteins extracted in 0.5 mM EDTA, 20 mM Tris.HCl, pH 8.0. The extracts were then derivatised for 60 min at room temperature and then subjected to LC-MS/MS analysis [71] with a Thermo Surveyor LC system coupled to a TSQ Quantum, triple quadrupole mass spectrometer (Thermo Scientific, Hemel Hempstead, UK). The 150 x 2.0 mm Stability 100 BS-C17 column (Hichrom, Reading, UK) was run at 45°C with 50% 15 mM ammonium acetate, pH 2.4 and 50% methanol at flow rates of 200 μl/minute. Total run times were 4 minutes. Electrospray ionisation was performed in positive ion mode with single reaction monitoring (SRM) of parent ions glu-glu (*m*/z 277—*m/z* 241), GSH-NEM (*m/z* 433—*m/z* 304) and GSSG (*m/z* 613—*m/z* 355). Quantification of GSH and GSSG concentrations was performed relative to calibration curves using Xcalibur software (Version 2.0.7.SP2), and the technical errors were less than 10%. The redox potential (ΔE) was calculated for cells at 30°C and pH 7.4 using the following equation [59].

$$\Delta E=-264-\frac{60.2}{2}\text{log}\left(\frac{{\left[GSH\right]}^{2}}{\left[GSSG\right]}\right)mV$$

### Transcript levels

RNA was prepared using published procedures [19,48] from mid-exponential *C*. *albicans* cells grown in YPDT and exposed to H_2_O_2_ for 10 minutes at the specified concentrations (above). cDNA was prepared using Superscript™ II Reverse Transcriptase (InVitrogen), and the qRT-PCR reactions were performed with a Light Cycler (Roche) using the primers and probes described in S8 Table. mRNA levels were calculated relative to the *ACT1* mRNA control [19,48]. We represent the SD of three independent experiments.

### Statistical analyses

The data is representative of observations from at least three independent replicate experiments. Means and standard deviations were calculated using at least triplicate measurements. Statistical significance was assessed using the t-test: *, p <0.05; **, p <0.01; ***, p <0.001.

### Model Construction

In this section we describe construction and integration of the model of the oxidative stress response in *C*. *albicans*, as well as the assumptions and hypothesis underlying this model.

First, the dynamics of the major redox components following H_2_O_2_ exposure are described using a system of ordinary differential equations, i.e.,

$$\frac{dX}{dt}=S\cdot v(X,K)$$

where *X* is the vector of species concentrations and *S* is a matrix of stoichiometric coefficients describing all model reactions. Reaction rates, defined by vector *v*, are a function of the relevant species concentrations *X*, and the associated vector of kinetic parameters, *K*. All of the biochemical reactions in our oxidative stress response model are listed in S1 Table. S2 Table presents the reactions rate equations and the associated kinetic parameters in our mathematical model. S3 and S4 Tables list the auxiliary variables and constants used in the model, respectively. The ordinary differential equations used in the model are given in S5 Table. Finally, S6 Table lists the initial concentrations of proteins, metabolites and enzymes that are considered in the model.

The model is partitioned into two compartments, namely the intracellular and extracellular spaces. The purpose of this compartmentalisation was to: (a) facilitate experimental validation, as some of our measurements involved culture media; and (b) minimize unnecessary complexity in the model. To account for the difference in volume between these compartments, the (H_2_O_2_^In^) and (H_2_O_2_^Ex^) mass balance equations were re-scaled according to their relative volumes. We assumed that the osmotically active volume of a single cell is 4.0 x 10^−14^ L (presuming spheres of radius r = 5 μm and a solid base volume of 41% similar to [50]). Also, the *C*. *albicans* cultures contained 3.5 x 10^7^ cells.mL^-1^ at the time of stress imposition, and therefore, the extracellular volume was estimated to be about 730 times larger than intracellular space.

#### Transporter module

This module describes the transport of H_2_O_2_ across the plasma membrane. Usually the extracellular medium does not contain any H_2_O_2_, and hence the initial concentration of extracellular H_2_O_2_ is assumed to be zero (i.e. H_2_O_2_^Ex^(t = 0) = 0). In our experimental setup, the application of extracellular oxidative stress is achieved by directly adding a specific concentration of H_2_O_2_ to the culture medium at a specific time point (say, *t = t*_*1*_). Therefore, the imposition of H_2_O_2_ stress upon *C*. *albicans* cells is modelled as a separate signal and is given by:

$$v_{1}(t)=\{\begin{array}{cc} S, & \begin{array}{cc} if & t=t_{1} \end{array}\\ 0, & else \end{array}$$

Since H_2_O_2_ is a secondary messenger involved in cell signalling [72], it is also assumed that *C*. *albicans* cells generate intracellular H_2_O_2_ (H_2_O_2_^In^) at a certain fixed rate, *k*_*4*_ (see Eq 4, in S2 Table). Furthermore, it is assumed that catalase (Cat1) plays a key role in regulating the basal steady state level of intracellular H_2_O_2_. Our experimental data suggest that inactivating this enzyme leads to accumulation of H_2_O_2_^In^, even under non-stress conditions (S2 Fig). Therefore together, intracellular H_2_O_2_ production, and Cat1-mediated detoxification are assumed to maintain a steady concentration of intracellular H_2_O_2_ under non-stress conditions (i.e. (H_2_O_2_^In^ (t = 0)) = (H_2_O_2_^SS^), where (H_2_O_2_^SS^) is the basal level of intracellular H_2_O_2_.

It has been established that H_2_O_2_ molecules undergo limited diffusion across the cytoplasmic membrane [26]. There is no detectable increase in extracellular H_2_O_2_ when *C*. *albicans* is grown in the absence of exogenous H_2_O_2_ (not shown). Nevertheless, it is assumed that there is an efflux of (H_2_O_2_^In^) into the medium when the net intracellular peroxide stress XS^In^ is greater than zero, where (XS^In^) = (H_2_O_2_^In^) − (H_2_O_2_^SS^). The limited diffusion rate of extracellular H_2_O_2_ across the plasma membrane is described by the following equation:

$$\frac{d[H_{2}O_{2}{}^{Ex}]}{dt}=v_{1}(t)+\left(\frac{\left(\left[XS^{In}]-[H_{2}O_{2}{}^{Ex}\right]\right)\times \left(10^{-3}\times P\times A\right)}{V_{m}}\right)-\left(\alpha^{H_{2}O_{2}}\times [H_{2}O_{2}{}^{Ex}]\right)$$

Where (H_2_O_2_^Ex^) is the concentration of peroxide in the extracellular media, (XS^In^) is the intracellular oxidative stress, *P* is the permeability coefficient of the plasma membrane, *A* is the surface area of the cell, *V*_*m*_ is the volume of the extracellular media and α^H2O2^ is rate of natural decay of H_2_O_2_. Similarly, the concentration of intracellular hydrogen peroxide is described as the sum of intracellular production, influx, efflux, scavenging of H_2_O_2_ and natural decay, respectively:

$$\frac{d[H_{2}O_{2}{}^{In}]}{dt}=v_{4}(t)+\left(\frac{\left(\left[H_{2}O_{2}{}^{Ex}]-[XS^{In}\right]\right)\times \left(10^{-3}\times P\times A\right)}{V_{os}}\right)-\sum_{i=5,8,10,15,18}v_{i}(t)-\left(\alpha^{H_{2}O_{2}}\times [H_{2}O_{2}{}^{In}]\right)$$

where *v*_*4*_ *(t) = k*_*4*_ is the constant rate of intracellular H_2_O_2_ production, *V*_*os*_ is the osmotically active volume of the cell and $\sum_{i=5,8,10,15,18}v_{i}(t)$ is the set of all reactions involved in the elimination of intracellular H_2_O_2_ (see S1 and S2 Tables).

#### Antioxidant module

This module is composed of four sub-modules. It is designed to describe the activity of the three different antioxidant systems of *C*. *albicans* and their NADPH source, namely, the pentose phosphate pathway (PPP). These sub-modules were constructed and parameterised so that their simulations were consistent with our experimental measurements and other published data.

*1*. *Catalase sub-module*: Catalase (Cat1) detoxifies H_2_O_2_ by converting it into water and oxygen. Our experiments show that H_2_O_2_ detoxification is primarily dependent on Cat1 (Fig 3a). As mentioned above, we have also assumed that Cat1 plays a major role in regulating basal intracellular peroxide levels (H_2_O_2_^SS^) under non-stress conditions.

The catalase mediated decomposition of intracellular H_2_O_2_ occurs in two steps via a ping-pong mechanism [73]. The rate of decay of H_2_O_2_ has been shown to be proportional to both (H_2_O_2_^In^) and (Cat1), and therefore the rate of elimination of intracellular peroxide by Cat1 is given by [73]:

$$v_{5}=k_{5}\times [H_{2}{}_{}O_{2}]\times [Cat1]$$

Where, k_5_ has the units of M^-1^sec^-1^ (Eq 5, S2 Table).

*2*. *Glutathione sub-module*: Glutathione (GSH) is the most abundant non-protein thiol in most eukaryotic cells [29,74]. Our measurements show that, under basal conditions, *C*. *albicans* cells contain about 9.67 mM GSH, and the ratio of GSH:GSSG is about 2:1 (Fig 1b and 1c). In order to include the strong basal redox activity of *C*. *albicans* in our model, we considered a simple second order mass action reaction that converts two molecules of GSH into a GSSG molecule at certain rate (Eq 6, S2 Table) [75]. Cells recycle GSSG back to GSH via glutathione reductase, which uses using NADPH. *C*. *albicans* contains a single glutathione reductase (Glr1), and the enzymatic conversion of GSSG to GSH was modelled in terms of random order two-substrate Michaelis-Menten kinetics (Eq 7, S2 Table) [30].

Together, the redox couple (GSH and GSSG) form the largest pool of reducing equivalents and therefore in the model they are considered to be the most important cellular redox buffer. As a result, at any given time, the redox state of this couple can be used as an indicator of the redox environment of the cell. The redox potential (ΔE) of the cell was calculated using Nernst equation and using GSH-GSSG half-cells, at 30°C and pH 7.4 is given by:

$$\Delta E=-264-\frac{60.2}{2}\text{log}\left(\frac{{\left[GSH\right]}^{2}}{\left[GSSG\right]}\right)mV$$

In other words, under basal conditions in the absence of oxidative stress, the redox potential of *C*. *albicans* cells is estimated to be about -202.23 mV [59].

When *C*. *albicans* cells are subjected to oxidative stress, GSH functions as the substrate of glutathione peroxidase which reduces excessive intracellular H_2_O_2_ [29]. While *C*. *albicans* contains three Gpx-like proteins, only one of them (Gpx1) is induced during H_2_O_2_ stress [32]. Therefore, we considered a single predominant glutathione peroxidase and called it Gpx. This Gpx-catalysed reaction is modelled as random two-substrate Michaelis-Menten kinetics (Eq 8, S2 Table) [30]. Also, protein thiols are vulnerable to oxidation. These are repaired by S-glutathionylation, and these S-glutathionylated proteins are eventually de-glutathionylated and recycled back to their native state using glutaredoxins and NADPH [41] (see "Protein-thiol Module" for more details).

Cellular GSH biosynthesis is regulated in response to oxidative stress [30,76]. Transcriptomic studies have shown that the *C*. *albicans* GSH biosynthetic gene, *GCS1*, is induced in a Cap1-dependent manner during oxidative stress [20]. Also, our measurements of GSH and GSSG suggest that cells produce more GSH in response to oxidative stress to promote H_2_O_2_ detoxification and protein protection. To reduce complexity, the GSH biosynthetic pathway is represented using a hypothetical mRNA called *GSH*.*mRNA* that is responsible for the production and maintenance of a steady state level of GSH and GSSG under basal conditions (Eq 47, S2 Table). During oxidative stress, it is assumed that the level of this hypothetical *GSH*.*mRNA* is increased in a Cap1-dependent manner, which in turn causes an increase in intracellular GSH levels (Eq 60, S2 Table).

*3*. *Thioredoxin sub-module*: Thioredoxins (Trx) regulate cellular reduction potential by participating in a diverse set of redox reactions [21,22,77,78]. Together with peroxiredoxin and thioredoxin reductase, they constitute the thioredoxin pathway, and maintain normal cellular function during oxidative stress. They detoxify H_2_O_2_, and they reduce cellular proteins that are oxidised to form disulphides [21,22,33,77,78]. Trx1 plays the major role in rescuing *C*. *albicans* cells during from oxidative stress [21]. Following H_2_O_2_ exposure, Tsa1 is oxidised by H_2_O_2_ to generate Tsa1^Ox^. Oxidised Tsa1 is then reduced by Trx1^Red^, which becomes oxidised to Trx1^Ox^. Trx1^Ox^ is then recycled back to its native form by Trr1^Red^. Finally, Trr1^Ox^ is converted back to Trr1^Red^ by accepting a proton from NADPH [21]. A similar redox-relay, starting from thioredoxin, reduces cellular proteins that are oxidised during peroxide stress (see "Protein-thiol Module" for more details). Unlike gluthathione, Trx1 exists in the native reduced state under stress-free conditions (Trx1^Red^) [21]. Since in the model redox status of the Trx1 depends upon both peroxiredoxin and thioredoxin reductase, auto-oxidation of Tsa1 or Trr1 is not considered in the model (i.e., k_17_ = 0). Therefore, both Tsa1 and Trr1 also exists in the native reduced state under stress-free conditions, i.e, as Tsa1^Red^ and Trr1^Red^ respectively.

The reactions of the thioredoxin system, including those involving protein di-thiol repair, were modelled using mass action kinetics, following the approach of Adimora *et al*. [24] (Eq 14–21, S2 Table).

*4*. *PPP sub-module*: The glutathione and thioredoxin systems rely upon the PPP for the NADPH required for H_2_O_2_ detoxification. In response to oxidative stress, *S*. *cerevisiae* cells induce NADPH production via a rapid increase in flux through the oxidative branch of the PPP, and subsequently via Yap1-mediated up-regulation of this oxidative branch of the PPP [65,36,37,38,39]. Both of these responses are important for the oxidative stress response in *S*. *cerevisiae*. Transcriptomic studies in *C*. *albicans* indicate that oxidative stress leads to a Cap1-dependent induction of oxidative branch genes of the PPP [40]. However, the rapid induction of NADPH production through the redirection of metabolic flux through this oxidative branch remains to be demonstrated experimentally. Nevertheless, both are included in our model.

In our model, the basal rate of NADPH production via the PPP is modelled as a simple zero-order mass action reaction (Eq 48, S2 Table). To reduce complexity, both the metabolic and Cap1-dependent induction of NADPH production was modelled abstractly in terms of Hill functions (Eqs 22 and 61, S2 Table). While the model considers the fast metabolic phase induction of NADPH to be dependent upon the (XS^In^) (to represent the rapid action of intracellular peroxide stress on the oxidative branch of the PPP), the relatively slower induction of the PPP genes is regarded to be proportional to the amount of activated Cap1 at any given time.

#### Protein thiol module

Many intracellular proteins contain cysteine thiols that are susceptible to oxidation. The oxidation state of these cysteine thiols can influence protein structure and functionality, and their vulnerability to oxidative damage depends on their local microenvironment. We adapted the approaches of Adimora *et al*. [24] and Jones *et al*. [33] to construct the protein thiol module to depict the redox-relay of cellular protein thiols during H_2_O_2_ stress.

Following an oxidative insult, protein mono-thiols are oxidised to form protein sulfenic acids (Pr.SOH), which then undergo S-glutathionylation to form mixed disulphides between protein and glutathione (Pr.SSG). Analogous to Adimora *et al*., our model assumes that Pr.SSG reduction is solely catalysed by glutaredoxins, with the use of glutathione and NADPH [24,41]. Of the four putative glutaredoxin genes in *C*. *albicans* (*GRX1*, *TTR1*, *GRX3* and *orf19*.*4150*) only *TTR1* is significantly induced during H_2_O_2_ stress [19]. Therefore, Ttr1 is used to represent glutaredoxin in the model. All reactions involving the formation and dissolution of mixed difulfides is modelled in terms of mass action kinetics [24] (Eqs 9–13, S2 Table) and the entire pool of protein thiols is assumed to exist in the reduced state under stress-free conditions.

During oxidative stress, protein di-thiols are oxidised to form protein disulphides (Pr.SSP). The model assumes that protein disulphides are recycled back to their native state via a series of redox reactions mediated by the thioredoxin system [24,33,34,35]. These were modelled in terms of simple mass action kinetics (Eq 14–16, S2 Table) [24]. Under non-stress conditions, these protein thiols are assumed to exist in their native reduced state.

#### Signalling and gene expression module

The signalling and gene expression module is composed of three sub-modules: (i) a Cap1 sub-module that describes the activation of Cap1 signalling during H_2_O_2_ stress; (ii) a Hog1 sub-module that characterises the onset of Hog1-dependent stress signalling during H_2_O_2_ exposure; and (iii) a gene expression sub-module that tracks the induction of the antioxidant and PPP genes during H_2_O_2_ stress.

*1*. *Cap1 sub-module*: Under normal conditions, Cap1 exists in its native-reduced-inactive state, (Cap1^N^) [21]. When *C*. *albicans* cells are subjected to H_2_O_2_ stress, Cap1 is oxidised and activated, by the action of Gpx3 and the Cap1-interacting protein Ybp1 [43]. Oxidised Cap1 (Cap1^A^) induces the transcription of oxidative stress genes, which encode components of the main antioxidant systems and the oxidative branch of the PPP [19,20,40,47]. Cap1 returns to its native-inactive state, thereby halting the activation of antioxidant and PPP genes following adaptation [21,43]. Recent findings suggest that Cap1 reduction is partially mediated by Trx1^Red^, as *trx1* mutants display prolonged Cap1 oxidation [21].

Our model includes a third state for Cap1: an inactive form of Cap1 (Cap1^I^). This third form was required to reproduce the observed dose-response data (Fig 4) [54]. In the model, Cap1^N^ is rapidly converted to Cap1^A^ through the indirect action of intracellular oxidative stress (XS^In^) [43]. The third inactive form of Cap1 (Cap1^I^) is assumed to be derived from the oxidised active form of Cap1 (Cap1^A^), and to be generated indirectly from the action of the H_2_O_2_ stress. The model also assumes that this third form of Cap1 is generated only when the H_2_O_2_^In^, and consequently the level of oxidative stress (XS), rises rise above a certain critical threshold (H_2_O_2_*) and (XS*) = (H_2_O_2_*)-(H_2_O_2_^In^), where (H_2_O_2_*) >>> H_2_O_2_^SS^). This assumption is based on experimental data that show a clear increase in the expression of Cap1 target genes when (H_2_O_2_^Ex^) ranges between 0–2 mM, and then a decrease in these targets beyond this threshold stress value. We have also assumed that the formation of Cap1^A^ and Cap1^I^ are reversible following H_2_O_2_ detoxification. To summarise, our model of Cap1 regulation can be represented as follows:

$${\text{Cap1}}^{\text{N}}\begin{array}{c} \overset{{}^{{\text{XS}}^{\text{In}}}}{\to }\\ \underset{{}^{{\text{Trx}}^{\text{Red}}}}{\leftarrow } \end{array}{\text{Cap1}}^{\text{A}}\begin{array}{c} \overset{{}^{{\text{XS}}^{*}}}{\to }\\ \underset{}{\leftarrow } \end{array}{\text{Cap1}}^{\text{I}}$$

Cap1 regulation is modelled in an abstract way. Under basal conditions, Cap1 is assumed to exist in its reduced, inactive state. Cap1 activation in response to H_2_O_2_ stress is described in terms of mass action kinetics, wherein the rate of Cap1 activation is directly proportional to both intracellular oxidative stress ((XS^In^)) and the amount of native inactive Cap1 (Cap1^N^) at any given time (Eq 23, S2 Table). Similarly, the rate of production of Cap1^I^ is modelled in terms of mass action kinetics, where Cap1^I^ production is proportional to both (Cap1^A^) and XS* (Eq 24, S2 Table). In both of these reactions, intracellular oxidative stress is considered as a modifier, and is not consumed during these processes. The thioredoxin-dependent reduction of Cap1^A^ and the reduction of Cap1^I^ are modelled using simple mass action kinetics (Eqs 25–26, S2 Table). Once again, thioredoxin is only regarded as modifier in these reactions.

*2*. *Hog1 sub-module*: The Hog1 MAPK module in *C*. *albicans* becomes activated in response to H_2_O_2_ stress. The Ssk2 MAPKKK phosphorylates the Pbs2 MAPKK, which in turn phosphorylates the Hog1 MAPK [6]. Hog1 then accumulates in the nucleus and activates key processes involved in oxidative stress adaptation [79]. Following adaptation, this MAPK cascade is deactivated by protein phosphatases. To represent this Hog1 MAPK system, we adapted the well-established model of Hog1 signalling in *S*. *cerevisiae* [50]. However, given the lack of clarity regarding phosphorelay signalling to Hog1 in *C*. *albicans* during oxidative stress [79], this was not included explicitly in our model. We modelled the MAPK module using mass action kinetics (Eqs 27 and 28, S2 Table) [50].

Previous models, which address Hog1 signalling in response to osmotic stress, include two main states for this MAPK: the native inactive form, and the phosphorylated active form of Hog1 (Hog1^N^ and Hog1^N^.PP, respectively). To adapt the model to represent Hog1 signalling during oxidative stress, we introduced two additional inactive forms of Hog1: a modified inactive non-phosphorylated form (Hog1^I^) and a modified inactive phosphorylated form (Hog1^I^.PP), in addition to the native reduced non-phosphorylated form (Hog1^N^) and the active phosphorylated form (Hog1^N^.PP). These additional forms were required because although *C*. *albicans* Hog1 is phosphorylated in response oxidative as well as osmotic stress [15], Hog1 mediates different outputs in response to these different stresses [19]. Also, given that Sty1 (the homologue of Hog1 in *S*. *pombe*) is oxidised following oxidative stress [51], we reasoned that *C*. *albicans* Hog1 might also be modified following H_2_O_2_ exposure. Our envisioned model of Hog1 regulation during oxidative stress can be schematically represented as follows:

$$\begin{array}{ccc} {\text{Hog1}}^{\text{N}} & \begin{array}{c} \overset{{}^{{\text{XS}}^{\text{In}}}}{\to }\\ \underset{{}^{{\text{Trx}}^{\text{Red}}}}{\leftarrow } \end{array} & {\text{Hog1}}^{\text{I}}\\ \begin{array}{cc} \text{Pbs2}•\text{PP} & ↓ \end{array}\begin{array}{cc} ↑ & \end{array} & & \begin{array}{ccccc} & & & & ↑ \end{array}\begin{array}{cc} ↓ & {}^{\text{Pbs2}•\text{PP}} \end{array}\\ {\text{Hog1}}^{\text{N}}\text{•PP} & \begin{array}{c} \overset{{}^{{\text{XS}}^{\text{In}}}}{\to }\\ \underset{{}^{{\text{Trx}}^{\text{Red}}}}{\leftarrow } \end{array} & {\text{Hog1}}^{\text{I}}\text{•PP} \end{array}$$

Our presumptions about the regulation of the modified states of Hog1 are as follows. First, upon exposure to H_2_O_2_ stress, both the native inactive and active forms of Hog1 (Hog1^N^ and Hog1^N^.PP) are modified to form Hog1^I^ and Hog1^I^.PP, respectively. Second, Trx1^Red^ limits modified Hog1 by reducing these forms of this SAPK to its native active or reduced state. Third, the active MAPKK (Pbs2.PP) phosphorylates Hog1^I^ in an analogous fashion to Hog1^N^. Fourth, the phosphatases that deactivate Hog1^N^.PP also dephosphorylate Hog1^I^.PP. The model further assumes that all modified forms of Hog1 are inactive. This generalised scheme of Hog1 regulation was designed to reflect available biological information about Hog1 regulation in *C*. *albicans*.

*3*. *Gene and protein expression sub-module*: This gene and protein expression sub-module was designed to track the Cap1- and Hog1-dependent gene responses of *C*. *albicans* cells to H_2_O_2_ stress. Cap1 activation leads to the induction of oxidative stress genes that include *CAT1*, *TSA1*, *TRR1*, *TRX1*, *GLR1*, *TTR1* and *CAP1* [19,20,40,47]. Cap1 also induces PPP (*TAL1*, *ZWF1*) and glutathione biosynthetic genes in response to H_2_O_2_ treatment (e.g., *GCS1*) [19,42]. Also, Hog1 induces *CAT1* in a Cap1-independent manner [19,42]. Therefore, in the model, genes were first considered to have a basal transcription rate in the absence of stress (Eqs 39–48, S2 Table). Then the Cap1- and Hog1-dependent induction of oxidative stress genes in response to H_2_O_2_ was modelled in terms of Hill functions (Eqs 52–62, S2 Table). To reduce complexity, the induction of GSH and NADPH production was modelled abstractly using Hill type functions.

The *SSK2*, *PBS2*, and *HOG1* transcripts are included in our model in addition to the above-mentioned mRNAs. These are assumed to be transcribed at a constant rate during non-stress and H_2_O_2_ stress conditions (Eq 49–51, S2 Table). Except for protein thiols, the model assumes that mRNAs are translated at a constant rate to maintain the corresponding pools of proteins. We model the rate of production of a given protein v_Pr_ by ν_Pr_ = r^*mRNA*^⋅[*mRNA*], where r^mRNA^ is the average translation rate of a given mRNA (Eqs 63–75, S2 Table). The model assumes a constant rate of production of the two different protein thiol pools (Eqs 9 and 4, S2 Table).

### Model simulations

Model simulations were performed using Matlab (R2012a). The governing set of ordinary differential equations was integrated using the stiff numerical ODE integrator (ode15s function). The model was initialised and parameterised using our own experimental data where possible, and these data were complemented with data from the literature (S2–S6 Tables). Due to the large number of ordinary differential equations, simultaneous parameter estimation with limited amount of experimental data is often intricate. Hence, consistency with available experimental data was ensured at each step of the model construction, as this approach has led to successful development of a number of predictive models that integrate a large number of cellular processes [43, 64, 74]. We have included detailed information about the estimation of each parameter and initial condition on S2–S6 Tables.

### Sensitivity analysis

In order to study the parameter sensitivity of the model we first generated 100 different sets of parameters by varying them simultaneously between 0.1 and 10 times their nominal parameter value (optimised one) according to a homogeneous random distribution, since a separate sensitivity analysis for each parameter was not feasible due to the large number of parameters in the model [80]. We varied 73 of the model parameters, and kept the rest of the parameters fixed which would alter the initial conditions of the model components, such as basal production and degradation rates. The model was simulated with each of those 100 parameter sets and relative errors (relative error = |x_E_-x_S_|/x_E_) were calculated by comparing the simulated concentration (x_S_) with their corresponding experimental values (x_E_) of extra-cellular H_2_O_2_, GSH, catalase and *TRR1* mRNA levels, at time points that best represent their stress response dynamics following a 5mM oxidative stress (10 min for catalase and *TRR1* mRNA levels, 15 min for extra-cellular H_2_O_2_, and 30 min for GSH). In this way we tested representative measures of all main three detoxifying systems as well as the decay of the oxidative stress signal.

This first analysis showed that the relative errors were close to 1 for most of the parameters combinations. Hence, in the next step, we set out to identify the most sensitive model parameters by pre-selecting groups of parameters playing a key role in the dynamics of the three main oxidative stress response systems, namely: (i) the H_2_0_2_ permeability rates k_2_ and k_3_; (ii) the reaction rates corresponding to the GSH sub-system (parameters of reactions v_6_ to v_8_); (iii) the ones corresponding to the TRX sub-system (parameters of reactions v_17_ to v_20_); (iv) the ones corresponding to Cap1 regulation (parameters of reactions v_23_ to v_26_); (v) the ones corresponding to Hog1 regulation (parameters of reactions v_27_ to v_38_); and finally, (vi) the parameters corresponding to mRNA induction (parameters of reactions v_39_ to v_51_). Then, in six new sets of simulations, those groups of parameters, (i) to (vi), were fixed separately according their nominal values, and all other parameters were varied according to a homogeneous random distribution between 0.1 and 10 times their nominal value, as before, thereby generating 100 new sets of parameter values for each case (i) to (vi).

### Ethics Statement

No ethical permissions were required for this work which involved no experimentation involving animals or human samples.

## Supporting Information

S1 Fig Decay of H_2_O_2_ in YPDT media. (PDF)

S2 Fig H_2_O_2_^Ex^ is sensitive to permeability of cellular membrane. (PDF)

S3 Fig Intracellular components are sensitive to mRNA induction rates. (PDF)

S1 Table List of biochemical reactions included the oxidative stress response model of *C*. *albicans*. (PDF)

S2 Table List of reaction rate equations for the oxidative stress model in *C*. *albicans*. (PDF)

S3 Table List of auxiliary variables used in the oxidative stress response model of *C*. *albicans*. (PDF)

S4 Table List of constants used in the oxidative stress response model of *C*. *albicans*. (PDF)

S5 Table List of ODEs of the oxidative stress response model of *C*. *albicans*. (PDF)

S6 Table List of model components of the oxidative stress response model of *C*. *albicans* and their initial conditions. (PDF)

S7 Table List of *C*. *albicans* strains used in this study. (PDF)

S8 Table qRT-PCR primers and probes used in this study. (PDF)
