# Supplementary material for: Integrative Model of Oxidative Stress Adaptation in the Fungal Pathogen Candida albicans
Source: PLoS One. 2015 Sep 14;10(9):e0137750. doi: 10.1371/journal.pone.0137750 (PMC4569071; doi:10.1371/journal.pone.0137750)
Supplement: S1 Fig — (PDF) [file pone.0137750.s001.pdf]

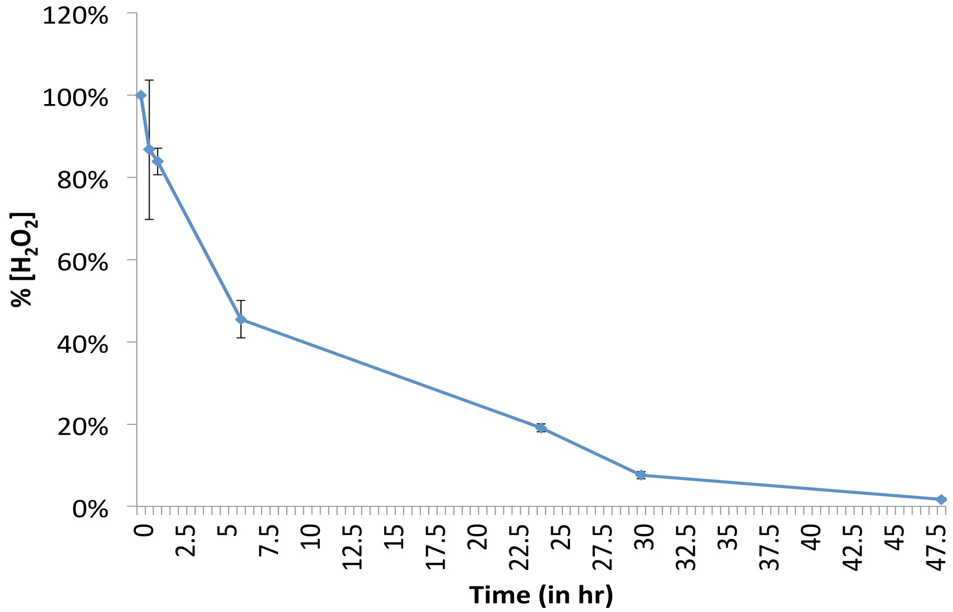

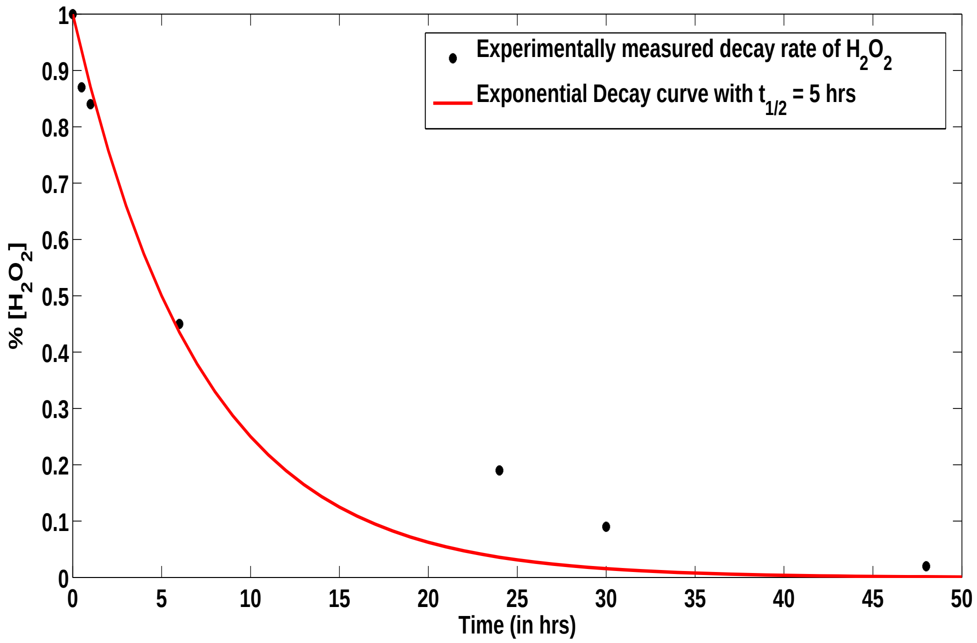

Figure S1. Decay of H_2O_2 in YPDT media: (Top) Experimentally measured decay of H_2O_2 in YPDT media (Tris buffered YPD: 2% w/v glucose, 2% w/v mycological peptone, 1% w/v yeast extract, 100 mM Tris.HCl, pH 7.4) after the addition of 5mM peroxide stress. (Bottom) Exponential decay process, with $t_{1/2} = 5$ hr, fitted to experimental data. The decay rate of H_2O_2 in YPDT medium is thus calculated to be 3.8508×10^{-5} s^{-1}.
