# Supplementary material for: Integrative Model of Oxidative Stress Adaptation in the Fungal Pathogen Candida albicans
Source: PLoS One. 2015 Sep 14;10(9):e0137750. doi: 10.1371/journal.pone.0137750 (PMC4569071; doi:10.1371/journal.pone.0137750)
Supplement: S2 Fig — (PDF) [file pone.0137750.s002.pdf]

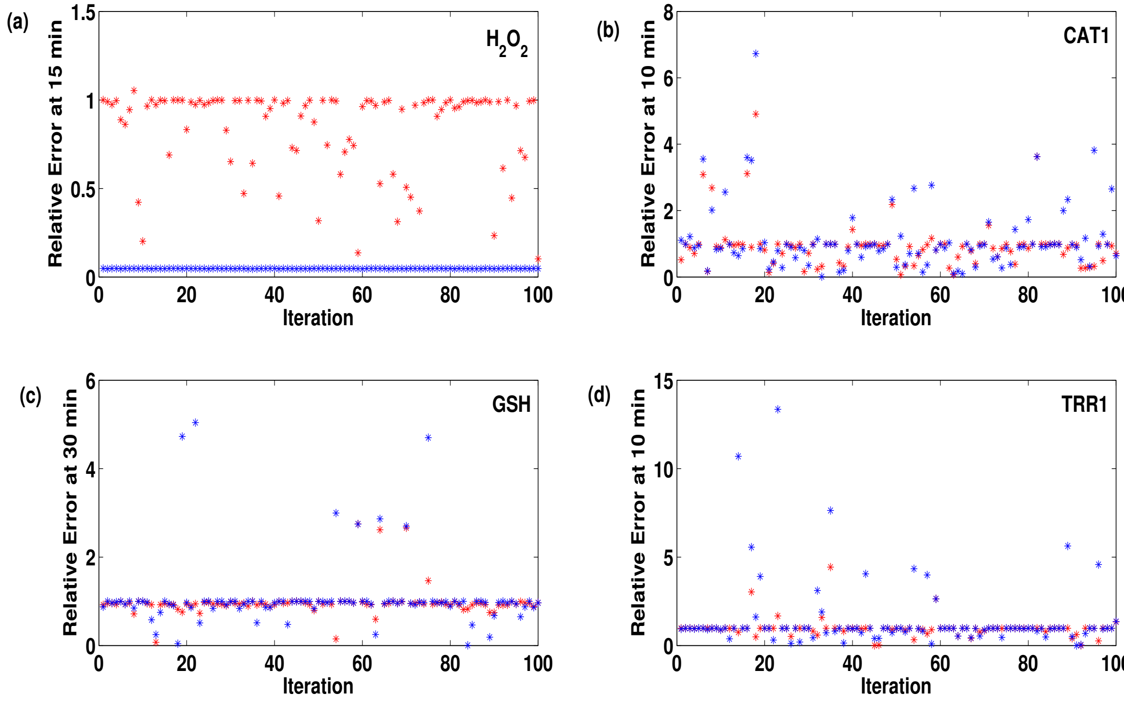

FIGURE S2. $H_2O_2^{Ex}$ is sensitive to permeability of cellular membrane: Relative errors for (a) $H_2O_2^{Ex}$ at 15 min, (b) catalase (CAT1) at 10 min, (c) GSH at 30 min, and (d) TRR1 mRNA levels at 10 min calculated for 100 different parameter sets. Red points correspond to the case when the set of 73 key model parameters were varied according a homogenous random distribution between 0.1 and 100 their nominal value, and blue points represent the case when only permeability rate was fixed to its nominal value, whereas the rest of the parameters were randomly varied as above. We see that by fixing the permeability rate the relative error in $H_2O_2^{Ex}$ substantially decreases, but not for the rest of the sensitivity measures.
