# Supplementary material for: Integrative Model of Oxidative Stress Adaptation in the Fungal Pathogen Candida albicans
Source: PLoS One. 2015 Sep 14;10(9):e0137750. doi: 10.1371/journal.pone.0137750 (PMC4569071; doi:10.1371/journal.pone.0137750)
Supplement: S3 Fig — (PDF) [file pone.0137750.s003.pdf]

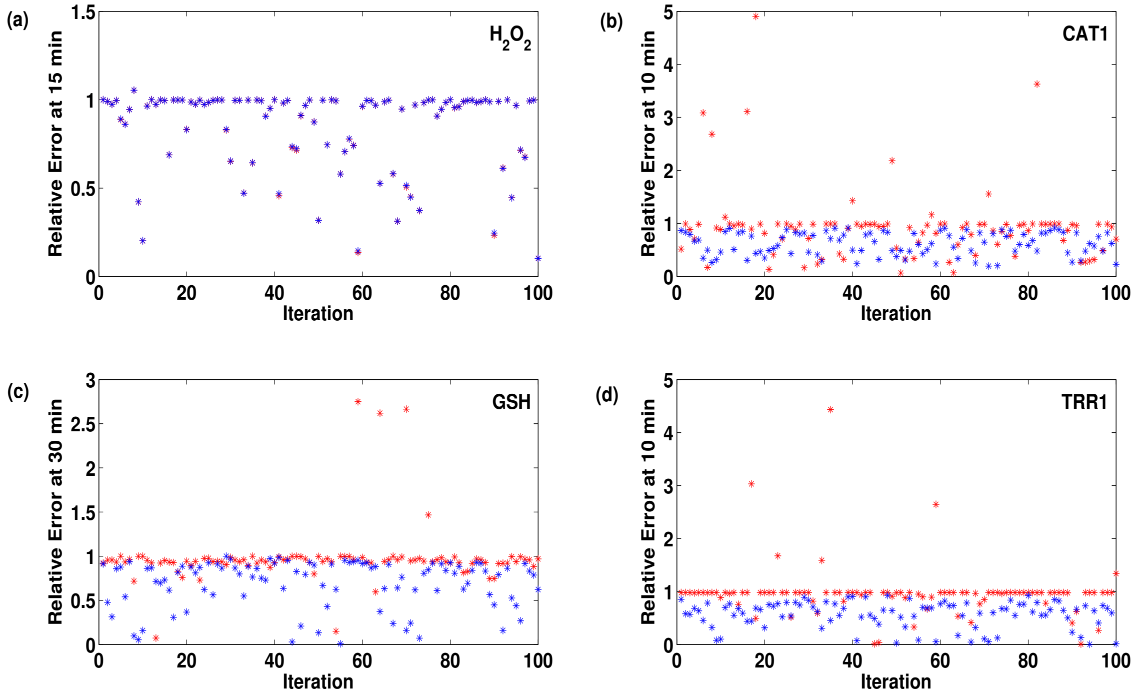

FIGURE S3. Intracellular components are sensitive to mRNA induction rates: Relative errors for (a) $H_2O_2^{Ex}$ at 15 min, (b) catalase (CAT1) at 10 min, (c) GSH at 30 min, and (d) TRR1 mRNA levels at 10 min calculated for 100 different parameter sets. Red points correspond to the case when the set of 73 model parameters were varied according a homogenous random distribution between 0.1 and 100 their nominal value, and blue points represent the case when only mRNA induction rates were fixed to their nominal values, whereas the rest of the parameters were randomly varied as above. We see that by fixing the mRNA induction rates the relative errors in CAT1, GSH and TRR1 decrease, but not for $H_2O_2^{Ex}$.
