# Supplementary material for: Integrative Model of Oxidative Stress Adaptation in the Fungal Pathogen Candida albicans
Source: PLoS One. 2015 Sep 14;10(9):e0137750. doi: 10.1371/journal.pone.0137750 (PMC4569071; doi:10.1371/journal.pone.0137750)
Supplement: S1 Table — (PDF) [file pone.0137750.s004.pdf]

Table S1: List of biochemical reactions included the oxidative stress response model of *C. albicans*.

No.	Reaction	Comment
1.	$\rightarrow H_2O_2^{Ex}$	Stress signal.
2.	$H_2O_2^{Ex} \rightarrow H_2O_2^{In}$	Limited diffusion of extra-cellular hydrogen peroxide ($H_2O_2^{Ex}$) into cytosol. The reaction is modelled as simple cell surface area dependent diffusion of $H_2O_2^{Ex}$.
3.	$XS^{In} \rightarrow H_2O_2^{Ex}$	Limited diffusion of excessive intra-cellular hydrogen peroxide ($H_2O_2^{In}$) out into the medium. Excessive $H_2O_2^{In}$, i.e., intracellular oxidative stress (XS^{In}), is defined as $XS^{In} = H_2O_2^{In} - H_2O_2^{ss}$, where $H_2O_2^{ss}$ is steady state value of $H_2O_2^{In}$ under normal condition. The reaction is modelled as simple cell surface area dependent diffusion of $H_2O_2^{In}$.
4.	$\rightarrow H_2O_2^{In}$	Basal rate of production of $H_2O_2^{In}$ that maintains $H_2O_2^{ss}$. It is modelled in terms of zero-order mass action reaction.
5.	$2H_2O_2^{In} \xrightarrow{Cat1} 2H_2O + O_2$	Enzymatic detoxification of excessive $H_2O_2^{In}$ by catalase ($Cta1$). This reaction is modelled in terms of mass action kinetics, as prescribed in literature.
6.	$2GSH \rightarrow GSSG$	Glutathione (GSH) converted to glutathione disulphide (GSSG) via cellular redox reactions under stress-free conditions. This reaction is modelled in terms of second-order mass action kinetics.
7.	$GSSG + NADPH + H^+ \xrightarrow{Glr1} 2GSH + NADP^+$	Enzymatic reduction of $GSSG$ by the enzyme $Glr1$. This reaction is modelled in terms of random two-substrate Michaelis-Menten kinetics.
8.	$XS^{In} + 2GSH \xrightarrow{Gpx} H_2O + GSSG$	Enzymatic detoxification of XS^{In} (i.e. excessive $H_2O_2^{In}$) by action of the Gpx. This reaction is modelled in terms of random two-substrate Michaelis-Menten reaction.
9.	$\rightarrow Pr.SH$	Basal rate of production of protein mono-thiols ($Pr.SH$). It is modelled in terms of zero-order mass action reaction.
10.	$Pr.SH + XS^{In} \rightarrow Pr.SOH + H_2O$	Oxidation of $Pr.SH$ to protein sulfenic acid ($Pr.SOH$) by XS^{In} (i.e. excessive $H_2O_2^{In}$). This reaction is modelled in terms of mass action kinetics.

11.	$Pr.SOH + GSH \rightarrow Pr.SSG + H_2O$	S-Glutathionylation of $Pr.SOH$ to mixed disulphide ($Pr.SSG$). The reaction is modelled using the law of mass action.
12.	$Pr.SSG + Ttr1^{Red} \rightarrow Pr.SH + Ttr1^{Ox}$	De-glutathionylation of $Pr.SSG$, via the action of glutaredxoin ($Ttr1^{Red}$), to native $Pr.SH$. The reaction is modelled using the law of mass action.
13.	$Ttr1^{Ox} + GSH \rightarrow Ttr1^{Red} + GSSG$	Reduction of oxidised glutaredoxin ($Ttr1^{Ox}$) by GSH. The reaction is modelled using the law of mass action.
14.	$\rightarrow Pr.(SH)_2$	Basal rate of production of protein di-thiols ($Pr.(SH)_2$). It is modelled in terms of zero-order mass action reaction.
15.	$Pr.(SH)_2 + XS^{In} \rightarrow Pr.SS + 2H_2O$	Oxidation of $Pr.(SH)_2$ by XS^{In} (i.e. excessive $H_2O_2^{In}$) and production of protein disulphide ($Pr.SS$). This reaction is modelled in terms of mass action kinetics.
16.	$Pr.SS + Trx1^{Red} \rightarrow Pr.(SH)_2 + Trx1^{Ox}$	Reduction of $Pr.SS$ by native thioredoxin ($Trx1^{Red}$). The reaction is modelled using the law of mass action.
17.	$Tsa1^{Red} \rightarrow Tsa1^{Ox}$	Cellular redox reaction causing oxidation of reduced peroxiredoxin ($Tsa1^{Red}$) under stress-free conditions. The reaction is modelled using the law of mass action.
18.	$XS^{In} + Tsa1^{Red} \rightarrow Tsa1^{Ox} + H_2O$	Detoxification of excessive $H_2O_2^{In}$ by $Tsa1^{Red}$. The reaction is modelled using the law of mass action.
19.	$Tsa1^{Ox} + Trx1^{Red} \rightarrow Tsa1^{Red} + Trx1^{Ox}$	Reduction of oxidised peroxyredoxin ($Tsa1^{Ox}$) by $Trx1^{Red}$. The reaction is modelled using the law of mass action.
20.	$Trx1^{Ox} + Trr1^{Red} \rightarrow Trx1^{Red} + Trr1^{Ox}$	Reduction of oxidised thioredoxin ($Trx1^{Ox}$) by the action of active thioredoxin reductase ($Trr1^{Red}$). The reaction is modelled using the law of mass action.
21.	$Trr1^{Ox} + NADPH + H^+ \rightarrow Trr1^{Red} + NADP^+$	Reduction of oxidised thioredoxin reductase ($Trr1^{Ox}$) by NADPH. The reaction is modelled using the law of mass action.
22.	$NADP^+ \xrightarrow{PPP/XS^{In}} NADPH$	Redirection of metabolic flux via PPP pathway under oxidative stress. NADPH induction is modelled in terms of Hill function.
23.	$Cap1^N \xrightarrow{XS^{In}} Cap1^A$	Oxidation and activation of the native transcription factor $Cap1^N$ to $Cap1^A$, by XS^{In}. The reaction is modelled using the law of mass action.

24.	$Cap1^A \xrightarrow{XS^*} Cap1^I$	Inactivation of active $Cap1^A$ to $Cap1^I$ under higher oxidative stress XS^* (i.e, when internal H_2O_2 levels go beyond a critical threshold). The reaction is modelled using using Hill type kinetics.
25.	$Cap1^I \rightarrow Cap1^A$	Conversion of $Cap1^I$ to $Cap1^A$. The reaction is modelled using the law of mass action.
26(a).	$Cap1^A \rightarrow Cap1^N$	Constitutive reduction of $Cap1^A$. The reaction is modelled using the law of mass action.
26(b).	$Cap1^A \xrightarrow{Trx1^{Red}} Cap1^N$	Reduction of $Cap1^A$ that is mediated by $Trx1^{Red}$. Here, it is assumed that $Trx1^{Red}$ only acts as a modifier and is not consumed during this reaction. The reaction is modelled using the law of mass action.
27.	$Ssk2 \xrightarrow{XS^{In}} Ssk2.P$	Phosphorylation and activation of MAPKKK $Ssk2$ by intracellular oxidative stress. The reaction is modelled using the law of mass action.
28.	$Ssk2.P \rightarrow Ssk2$	De-phosphorylation and de-activation of MAPKKK $Ssk2$ by phophotases. The reaction is modelled using the law of mass action.
29.	$Pbs2 \xrightarrow{Ssk2.P} Pbs2.PP$	Phosphorylation and activation of MAPKK $Pbs2$ by $Ssk2.P$. It is assumed that $Ssk2.P$ is not consumed during this process and only acts as a modifier. The reaction is modelled using the law of mass action.
30.	$Pbs2.PP \rightarrow Pbs2$	De-phosphorylation and de-activation of $Pbs2$ via phosphatases. The reaction is modelled using the law of mass action.
31.	$Hog1^N \xrightarrow{Pbs2.PP} Hog1^N.PP$	Phosphorylation and activation of native SAPK $Hog1$ by $Pbs2.PP$. It is assumed that $Pbs2.PP$ is not consumed in this process. The reaction is modelled using the law of mass action.
32.	$Hog1^N.PP \rightarrow Hog1^N$	De-Phosphorylation and de-activation of $Hog1^N.PP$ by phosphotases. The reaction is modelled using the law of mass action.
33.	$Hog1^I \xrightarrow{Trx1^{Red}} Hog1^N$	Conversion of $Hog1^N$ by $Trx1^{Red}$. $Trx1^{Red}$ is a modifier and is not turned in to $Trx1^{Ox}$ during this process. The reaction is modelled as mass action kinetics.
34.	$Hog1^N \xrightarrow{XS^{In}} Hog1^I$	Inactivation of $Hog1^N$ under oxidative stress. Once again, XS^{In} is considered as a modifier. The reaction is modelled using the law of mass action.

35.	$Hog1^I \xrightarrow{Pbs2.PP} Hog1^I.PP$	Phosphorylation of inactive $Hog1$ by $Pbs2.PP$. $Pbs2.PP$ is considered to be a modifier and is not consumed in this process. The reaction is modelled as mass action reaction.
36.	$Hog1^I.PP \rightarrow Hog1^I$	De-phosphorylation of $Hog1^I.PP$ by phosphatases. The reaction is modelled using mass action law.
37.	$Hog1^I.PP \xrightarrow{Trx1^{Red}} Hog1^N.PP$	Conversion of $Hog1^I.PP$ to $Hog1^N.PP$. The reaction is influenced by the modifier $Trx1^{Red}$ which itself is not consumed in the process. It is modelled using the mass action law.
38.	$Hog1^N.PP \xrightarrow{XS^{In}} Hog1^I.PP$	Conversion of $Hog1^N.PP$ to $Hog1^I.PP$ during oxidative stress. The reaction is modelled in term of mass action reaction.
39*.	$\rightarrow CAT1$	Basal rate of production of CAT1 mRNA. It is modelled in terms of zero-order reaction.
40*.	$\rightarrow CAP1$	Basal rate of production of CAP1 mRNA. It is modelled in terms of zero-order reaction.
41*.	$\rightarrow GPX$	Basal rate of production of GPX mRNA. It is modelled in terms of zero-order reaction.
42*.	$\rightarrow GLR1$	Basal rate of production of GLR1 mRNA. It is modelled in terms of zero-order reaction.
43*.	$\rightarrow TTR1$	Basal rate of production of TTR1 mRNA. It is modelled in terms of zero-order reaction.
44*.	$\rightarrow TSA1$	Basal rate of production of TSA1 mRNA. It is modelled in terms of zero-order reaction.
45*.	$\rightarrow TRX1$	Basal rate of production of TRX1 mRNA. T It is modelled in terms of zero-order reaction.
46*.	$\rightarrow TRR1$	Basal rate of production of TRR1 mRNA. It is modelled in terms of zero-order reaction..
47*.	$\rightarrow GSH.mRNA$	Basal rate of production of hypothetical GSH mRNA. It is modelled in terms of zero-order reaction.
48*.	$\rightarrow NADPH.mRNA$	Basal rate of production of hypothetical NADPH mRNA. It is modelled in terms of zero-order reaction.
49.	$\rightarrow SSK2$	Basal rate of production of SSK2 mRNA. It is modelled in terms of zero-order reaction.
50.	$\rightarrow PBS2$	Basal rate of production of PBS2 mRNA. It is modelled in terms of zero-order reaction.

51.	$\rightarrow HOG1$	Basal rate of production of HOG1 mRNA. It is modelled in terms of zero-order reaction.
52.	$Cap1^A \rightarrow CAT1$	Induction of $CAT1$ mRNA by active $Cap1$. The biochemical reaction is modelled in terms of Hill function.
53.	$Cap1^A \rightarrow CAP1$	Induction of $CAP1$ mRNA by active $Cap1$. The biochemical reaction is modelled in terms of Hill function.
54.	$Cap1^A \rightarrow GPX$	Induction of GPX mRNA by active $Cap1$. The biochemical reaction is modelled in terms of Hill function.
55.	$Cap1^A \rightarrow GLR1$	Induction of $GLR1$ mRNA by active $Cap1$. The biochemical reaction is modelled in terms of Hill function.
56.	$Cap1^A \rightarrow TTR1$	Induction of $TTR1$ mRNA by active $Cap1$. The biochemical reaction is modelled in terms of Hill function.
57.	$Cap1^A \rightarrow TSA1$	Induction of $TSA1$ mRNA by active $Cap1$. The biochemical reaction is modelled in terms of Hill function.
58.	$Cap1^A \rightarrow TRX1$	Induction of $TRX1$ mRNA by active $Cap1$. The biochemical reaction is modelled in terms of Hill function.
59.	$Cap1^A \rightarrow TRR1$	Induction of $TRR1$ mRNA by active $Cap1$. The biochemical reaction is modelled in terms of Hill function.
60.	$Cap1^A \rightarrow GSH.mRNA$	Induction of genes in glutathione synthesis pathway modelled as induction in hypothetical $GSH.mRNA$. This induction is modelled in terms of Hill function.
61.	$Cap1^A \rightarrow NADPH.mRNA$	Induction of genes in the oxidative branch of PPP is modelled as induction in hypothetical $NADPH.mRNA$. This induction is modelled in terms of Hill function.
62.	$Hog1^N.PP \rightarrow CAT1$	Induction of $CAT1$ mRNA by $Hog1^N.PP$. The biochemical reaction is modelled in terms of Hill function.
63.	$CAT1 \rightarrow Cat1$	Translation of $CAT1$ mRNA. This reaction is modelled using mass action law.
64.	$CAP1 \rightarrow Cap1^N$	Translation of $CAP1$ mRNA. It is assumed that the translation only produces the native form of $Cap1$ protein. This reaction is modelled using mass action law.

65.	$GPX \rightarrow Gpx$	Translation of GPX mRNA. This reaction is modelled using mass action law.
66.	$GLR1 \rightarrow Glr1$	Translation of $GLR1$ mRNA. This reaction is modelled using mass action law.
67.	$TTR1 \rightarrow Ttr1^{Red}$	Translation of $TTR1$ mRNA. It is assumed that the translation only produces the reduced form of $Ttr1$ protein. This reaction is modelled using mass action law.
68.	$TSA1 \rightarrow Tsa1^{Red}$	Translation of $TSA1$ mRNA. It is assumed that the translation only produces the reduced form of $Tsa1$ protein.This reaction is modelled using mass action law.
69.	$TRX1 \rightarrow Trx1^{Red}$	Translation of $TRX1$ mRNA. It is assumed that the translation only produces the reduced form of $Trx1$ protein.This reaction is modelled using mass action law.
70.	$TRR1 \rightarrow Trr1^{Red}$	Translation of $TRR1$ mRNA. It is assumed that the translation only produces the reduced form of $Trr1$ protein.This reaction is modelled using mass action law.
71.	$GSH.mRNA \rightarrow GSH$	Translation of hypothetical GSH mRNA. It is assumed that the translation only produces the reduced form of GSH. This reaction is modelled using mass action law.
72.	$NADPH.mRNA \rightarrow NADPH$	Translation of hypothetical $NADPH$ mRNA.This reaction is modelled using mass action law.
73.	$SSK2 \rightarrow Ssk2$	Translation of $SSK2$ mRNA. It is assumed that the translation only produces the unphosphorylated form of $Ssk2$. This reaction is modelled using mass action law.
74.	$PBS2 \rightarrow Pbs2$	Translation of $PBS2$ mRNA. It is assumed that the translation only produces the unphosphorylated form of $Pbs2$. This reaction is modelled using mass action law.
75.	$HOG1 \rightarrow Hog1^{N}$	Translation of $HOG1$ mRNA. It is assumed that the translation only produces the unphosphorylated native form of $Hog1$. This reaction is modelled using mass action law.
76.	$H_2O_2^{Ex} \rightarrow \phi$	Natural first order decay of external H_2O_2.
77.	$H_2O_2^{In} \rightarrow \phi$	Natural first order decay of internal H_2O_2.
78.	$Cat1 \rightarrow \phi$	First order decay of $Cat1$.

79.	$GSH \rightarrow \phi$	First order decay of GSH.
80.	$GSSG \rightarrow \phi$	First order decay of $GSSG$.
81.	$Gpx \rightarrow \phi$	First order decay of Gpx.
82.	$Glr1 \rightarrow \phi$	First order decay of $Glr1$.
83.	$Ttr1^{Red} \rightarrow \phi$	First order decay of $Ttr1^{Red}$.
84.	$Ttr1^{Ox} \rightarrow \phi$	First order decay of $Ttr1^{Ox}$.
85.	$Pr.SH \rightarrow \phi$	First order decay of $Pr.SH$.
86.	$Pr.SOH \rightarrow \phi$	First order decay of $Pr.SOH$.
87.	$Pr.SSG \rightarrow \phi$	First order decay of $Pr.SSG$.
88.	$Pr.(SH)_2 \rightarrow \phi$	First order decay of $Pr.(SH)_2$.
89.	$Pr.SS \rightarrow \phi$	First order decay of $Pr.SS$.
90.	$Tsa1^{Red} \rightarrow \phi$	First order decay of $Tsa1^{Red}$.
91.	$Tsa1^{Ox} \rightarrow \phi$	First order decay of $Tsa1^{Ox}$.
92.	$Trx1^{Red} \rightarrow \phi$	First order decay of $Trx1^{Red}$.
93.	$Trx1^{Ox} \rightarrow \phi$	First order decay of $Trx1^{Ox}$.
94.	$Trr1^{Red} \rightarrow \phi$	First order decay of $Trr1^{Red}$.
95.	$Trr1^{Ox} \rightarrow \phi$	First order decay of $Trr1^{Ox}$.
96.	$NADPH \rightarrow \phi$	First order decay of $NADPH$.
97.	$Cap1^{N} \rightarrow \phi$	First order decay of $Cap1^{N}$.
98.	$Cap1^{A} \rightarrow \phi$	First order decay of $Cap1^{A}$.
99.	$Cap1^{I} \rightarrow \phi$	First order decay of $Cap1^{I}$.
100.	$Ssk2 \rightarrow \phi$	First order decay of $Ssk2$.
101.	$Ssk2.P \rightarrow \phi$	First order decay of $Ssk2.P$.
102.	$Pbs2 \rightarrow \phi$	First order decay of $Pbs2$.
103.	$Pbs2.PP \rightarrow \phi$	First order decay of $Pbs2.PP$.
104.	$Hog1^{N} \rightarrow \phi$	First order decay of $Hog1^{N}$.
105.	$Hog1^{N}.PP \rightarrow \phi$	First order decay of $Hog1^{N}.PP$.
106.	$Hog1^{I} \rightarrow \phi$	First order decay of $Hog1^{I}$.

107.	$Hog1^I.PP \rightarrow \phi$	First order decay of $Hog1^I.PP$.
108.	$CAT1 \rightarrow \phi$	First order decay of $CAT1$.
109.	$CAP1 \rightarrow \phi$	First order decay of $CAP1$.
110.	$GPX \rightarrow \phi$	First order decay of GPX.
111.	$GLR1 \rightarrow \phi$	First order decay of $GLR1$.
112.	$TTR1 \rightarrow \phi$	First order decay of $TTR1$.
113.	$TSA1 \rightarrow \phi$	First order decay of $TSA1$.
114.	$TRX1 \rightarrow \phi$	First order decay of $TRX1$.
115.	$TRR1 \rightarrow \phi$	First order decay of $TRR1$.
116.	$SSK2 \rightarrow \phi$	First order decay of $SSK2$.
117.	$PBS2 \rightarrow \phi$	First order decay of $PBS2$.
118.	$HOG1 \rightarrow \phi$	First order decay of $HOG1$.
119.	$GSH.mRNA \rightarrow \phi$	First order decay of the hypothetical GSH mRNA.
120.	$NADPH.mRNA \rightarrow \phi$	First order decay of the hypothetical $NADPH$ mRNA.

* Reactions 39-48 represent the transcription process where the mRNA molecules are synthesised from the DNA molecules under stress free conditions.
