# Supplementary material for: Integrative Model of Oxidative Stress Adaptation in the Fungal Pathogen Candida albicans
Source: PLoS One. 2015 Sep 14;10(9):e0137750. doi: 10.1371/journal.pone.0137750 (PMC4569071; doi:10.1371/journal.pone.0137750)
Supplement: S2 Table — (PDF) [file pone.0137750.s005.pdf]

Table S2: List of reaction rate equations for the oxidative stress model in *C.albicans*. The units of the kinetic rate constants are $M \cdot s^{-1}$, $s^{-1}$, $M^{-1} \cdot s^{-1}$ or $M^{-2} \cdot s-2$ for $0^{th}$, $1^{st}$ and $2^{nd}$ order mass action reaction respectively.

| No. | Rate Equation | Rate Constant | Reference |
|---|---|---|---|
| 1. | $v_1(t) = S(t)$ | $S(t)$ - See Table S3 | |
| 2. | $v_2(t) = k_2 \times H_2O_2^{Ex}(t) \times a \times A$ | $k_2 = P = 3.5 \times 10^{-5}$ | Fitted |
| | | $a$ - See Table S4 | |
| | | $A$ - See Table S4 | |
| 3. | $v_3(t) = k_3 \times XS^{In}(t) \times a \times A$ | $k_3 = k_2 = P = 3.5 \times 10^{-5}$ | Fitted |
| | | $XS^{In}(t)$ - See Table S3 | |
| 4. | $v_4(t) = k_4$ | $k_4 = 2.1928 \times 10^{-7}$ | Fitted |
| 5. | $v_5(t) = k_5 \times Cat1(t) \times H_2O_2^{In}(t)$ | $k_5 = 3.4 \times 10^7$ | [1] |
| 6. | $v_6(t) = k_6 \times GSH(t) \times GSH(t)$ | $k_6 = 2.4266 \times 10^{-2}$ | Fitted |
| 7. | $v_7(t) = \frac{k_7 \times Glr1(t) \times GSSG(t) \times NADPH(t)}{\left(K_{M7}^{GSSG} + GSSG(t)\right) \times \left(K_{M7}^{NADPH} + NADPH(t)\right)}$ | $k_7 = 9 \times 10^{-1}$ | Fitted |
| | | $K_{M7}^{GSSG} = 0.2625 \times 10^{-3}$ | [2] |
| | | $K_{M7}^{NADPH} = 0.0323 \times 10^{-3}$ | [2] |
| 8. | $v_8(t) = \frac{k_8 \times Gpx(t) \times GSH(t) \times XS^{In}(t)}{\left(K_{M8}^{GSH} + GSH(t)\right) \times \left(K_{M8}^{H_2O_2} + XS(t)\right)}$ | $k_8 = 578$ | [3] |
| | | $K_{M8}^{GSH} = 1 \times 10^{-2}$ | Fitted |
| | | $K_{M8}^{H_2O_2} = 141 \times 10^{-6}$ | Fitted |
| 9. | $v_9(t) = k_9$ | $k_9 = 3.2777 \times 10^{-8}$ | Fitted |
| 10. | $v_{10}(t) = k_{10} \times Pr.SH(t) \times XS^{In}(t)$ | $k_{10} = 1 \times 10^4$ | [1] |
| 11. | $v_{11}(t) = k_{11} \times Pr.SOH(t) \times GSH(t)$ | $k_{11} = 1.2 \times 10^5$ | [1] |
| 12. | $v_{12}(t) = k_{12} \times Pr.SSG(t) \times Ttr1^{Red}(t)$ | $k_{12} = 9.1 \times 10^4$ | [1] |
| 13. | $v_{13}(t) = k_{13} \times Ttr1^{Ox}(t) \times GSH(t)$ | $k_{13} = 3.7 \times 10^4$ | [1] |
| 14. | $v_{14}(t) = k_{14}$ | $k_{14} = k_9 = 2.6866 \times 10^{-7}$ | Fitted |
| 15. | $v_{15}(t) = k_{15} \times Pr.(SH)_2(t) \times XS^{In}(t)$ | $k_{15} = 5 \times 10^1$ | Fitted |
| 16. | $v_{16}(t) = k_{16} \times Pr.SS(t) \times Trx1^{Red}(t)$ | $k_{16} = 1 \times 10^5$ | [1] |
| 17. | $v_{17}(t) = k_{17} \times Tsa1^{Red}(t)$ | $k_{17} = 0$ | Assumed |
| 18. | $v_{18}(t) = k_{18} \times Tsa1^{Red}(t) \times XS^{In}(t)$ | $k_{18} = 4 \times 10^7$ | [1] |
| 19. | $v_{19}(t) = k_{19} \times Tsa1^{Ox}(t) \times Trx1^{Red}(t)$ | $k_{19} = 1 \times 10^1$ | Fitted |
| 20. | $v_{20}(t) = k_{20} \times Trx1^{Ox}(t) \times Trr1^{Red}(t)$ | $k_{20} = 2 \times 10^1$ | Fitted |
| 21. | $v_{21}(t) = k_{21} \times Trr1^{Ox}(t) \times NADPH(t)$ | $k_{21} = 1 \times 10^2$ | Fitted |
| 22. | $v_{22}(t) = \frac{k_{22} \times XS^{In}(t)^{n22}}{K_{22}^{n22} + XS(t)^{n22}}$ | $k_{22} = 2.9619 \times 10^{-8}$ | Fitted |
| | | $n_{22} = 1$ | Fitted |

| | | | |
|---|---|---|---|
| | | $K_{22} = 1 \times 10^{-9}$ | Fitted |
| 23. | $v_{23}(t) = k_{23} \times Cap1^{N}(t) \times XS^{In}(t)$ | $k_{23} = 2.3258 \times 10^{6}$ | Fitted |
| 24. | $v_{24}(t) = k_{24} \times Cap1^{A}(t) \times XS^{*}(t)$ | $k_{24} = 1.1629 \times 10^{9}$<br>$XS^{*}(t)$ - See Table. S3 | Fitted |
| 25. | $v_{25}(t) = k_{25} \times Cap1^{I}(t)$ | $k_{25} = 5 \times 10^{-4}$ | Fitted |
| 26. | $v_{26}(t) = Cap1^{A}(t) \times (k^{a}_{26} + k^{b}_{26} \times Trx1^{Red}(t))$ | $k^{a}_{26} = 5$<br>$k^{b}_{26} = 1 \times 10^{3}$ | Fitted<br>Fitted |
| 27. | $v_{27}(t) = k_{27} \times XS^{In} \times Ssk2(t)$ | $k_{27} = 1.1629 \times 10^{4}$ | Fitted |
| 28. | $v_{28}(t) = k_{28} \times Ssk2.P(t)$ | $k_{28} = 1.359 \times 10^{-2}$ | Fitted |
| 29. | $v_{29}(t) = k_{29} \times Pbs2(t) \times Ssk2.P(t)$ | $k_{29} = 2.19 \times 10^{6}$ | Fitted |
| 30. | $v_{30}(t) = k_{30} \times Pbs2.PP(t)$ | $k_{30} = 2.007 \times 10^{-2}$ | Fitted |
| 31. | $v_{31}(t) = k_{31} \times Hog1^{N}(t) \times Pbs2.PP(t)$ | $k_{31} = 6.493 \times 10^{6}$ | Fitted |
| 32. | $v_{32}(t) = k_{32} \times Hog1^{N} \cdot PP(t)$ | $k_{32} = 5.906 \times 10^{-2}$ | Fitted |
| 33. | $v_{33}(t) = k_{33} \times Hog1^{I}(t) \times Trx1^{Red}(t)$ | $k_{33} = 5 \times 10^{-5}$ | Fitted |
| 34. | $v_{34}(t) = k_{34} \times Hog1^{N}(t) \times XS^{In}(t)$ | $k_{34} = 5 \times 10^{2}$ | Fitted |
| 35. | $v_{35}(t) = k_{35} \times Hog1^{I}(t) \times Pbs2.PP(t)$ | $k_{35} = 6.493 \times 10^{6}$ | Fitted |
| 36. | $v_{36}(t) = k_{36} \times Hog1^{I}.PP(t)$ | $k_{36} = 5.906 \times 10^{-2}$ | Fitted |
| 37. | $v_{37}(t) = k_{37} \times Hog1^{I}.PP(t) \times Trx1^{Red}(t)$ | $k_{37} = 6.493 \times 10^{6}$ | Fitted |
| 38. | $v_{38}(t) = k_{38} \times Hog1^{N}.PP(t) \times XS^{In}(t)$ | $k_{38} = 5.906 \times 10^{-2}$ | Fitted |
| 39. | $v_{39}(t) = k_{39}$ | $k_{39} = \beta^{CAT1} = 2.9619 \times 10^{-13}$ | [4] |
| 40. | $v_{40}(t) = k_{40}$ | $k_{40} = \beta^{CAP1} = 5.3601 \times 10^{-14}$ | [4] |
| 41. | $v_{41}(t) = k_{41}$ | $k_{41} = \beta^{GPX} = 2.3805 \times 10^{-13}$ | [4] |
| 42. | $v_{42}(t) = k_{42}$ | $k_{42} = \beta^{GLR1} = 1.0792 \times 10^{-13}$ | [4] |
| 43. | $v_{43}(t) = k_{43}$ | $k_{43} = \beta^{TTR1} = 4.4390 \times 10^{-13}$ | [4] |
| 44. | $v_{44}(t) = k_{44}$ | $k_{44} = \beta^{TSA1} = 1.8694 \times 10^{-12}$ | [4] |
| 45. | $v_{45}(t) = k_{45}$ | $k_{45} = \beta^{TRX1} = 5.4929 \times 10^{-13}$ | [4] |
| 46. | $v_{46}(t) = k_{46}$ | $k_{46} = \beta^{TRR1} = 3.0006 \times 10^{-13}$ | [4] |
| 47. | $v_{47}(t) = k_{47}$ | $k_{47} = \beta^{GSHmRNA} = 2.9619 \times 10^{-13}$ | [4] |
| 48. | $v_{48}(t) = k_{48}$ | $k_{48} = \beta^{NADPHmRNA} = 2.9619 \times 10^{-13}$ | [4] |
| 49. | $v_{49}(t) = k_{49}$ | $k_{49} = \beta^{SSK2} = 1.0161 \times 10^{-13}$ | [4] |
| 50. | $v_{50}(t) = k_{50}$ | $k_{50} = \beta^{PBS2} = 8.2790 \times 10^{-14}$ | [4] |
| 51. | $v_{51}(t) = k_{51}$ | $k_{51} = \beta^{HOG1} = 1.7012 \times 10^{-13}$ | [4] |
| 52. | $v_{52}(t) = \frac{k_{52} \times [Cap1^{A}]^{n_{52}}}{(K_{52})^{n_{52}} + [Cap1^{A}(t)]^{n_{52}}}$ | $k_{52} = \beta^{CTA1}_{Cap1} = 4.2398 \times 10^{-11}$<br>$n_{52} = 1$ | Fitted<br>Fitted |

| | | | |
|---|---|---|---|
| | | $K_{52} = 7 \times 10^{-8}$ | Fitted |
| 53. | $v_{53}(t) = \frac{k_{53} \times [Cap1^A]^{n_{53}}}{(K_{53})^{n_{53}} + [Cap1^A(t)]^{n_{53}}}$ | $k_{53} = \beta^{CAP1}_{Cap1} = 2.9009 \times 10^{-12}$<br>$n_{53} = 1$<br>$K_{53} = 7 \times 10^{-8}$ | Fitted<br>Fitted<br>Fitted |
| 54. | $v_{54}(t) = \frac{k_{54} \times [Cap1^A]^{n_{54}}}{(K_{54})^{n_{54}} + [Cap1^A(t)]^{n_{54}}}$ | $k_{54} = \beta^{GPX}_{Cap1} = 5.1062 \times 10^{-11}$<br>$n_{54} = 1$<br>$K_{54} = 7 \times 10^{-8}$ | Fitted<br>Fitted<br>Fitted |
| 55. | $v_{55}(t) = \frac{k_{55} \times [Cap1^A]^{n_{55}}}{(K_{55})^{n_{55}} + [Cap1^A(t)]^{n_{55}}}$ | $k_{55} = \beta^{GLR1}_{Cap1} = 2.0353 \times 10^{-11}$<br>$n_{55} = 1$<br>$K_{55} = 7 \times 10^{-8}$ | Fitted<br>Fitted<br>Fitted |
| 56. | $v_{56}(t) = \frac{k_{56} \times [Cap1^A]^{n_{56}}}{(K_{56})^{n_{56}} + [Cap1^A(t)]^{n_{56}}}$ | $k_{56} = \beta^{TTR1}_{Cap1} = 5.1271 \times 10^{-11}$<br>$n_{56} = 1$<br>$K_{56} = 7 \times 10^{-8}$ | Fitted<br>Fitted<br>Fitted |
| 57. | $v_{57}(t) = \frac{k_{57} \times [Cap1^A]^{n_{57}}}{(K_{57})^{n_{57}} + [Cap1^A(t)]^{n_{57}}}$ | $k_{57} = \beta^{TSA1}_{Cap1} = 2.8041 \times 10^{-10}$<br>$n_{57} = 1$<br>$K_{57} = 7 \times 10^{-8}$ | Fitted<br>Fitted<br>Fitted |
| 58. | $v_{58}(t) = \frac{k_{58} \times [Cap1^A]^{n_{58}}}{(K_{58})^{n_{58}} + [Cap1^A(t)]^{n_{58}}}$ | $k_{58} = \beta^{TRX1}_{Cap1} = 3.9549 \times 10^{-11}$<br>$n_{58} = 1$<br>$K_{58} = 7 \times 10^{-8}$ | Fitted<br>Fitted<br>Fitted |
| 59. | $v_{59}(t) = \frac{k_{59} \times [Cap1^A]^{n_{59}}}{(K_{59})^{n_{59}} + [Cap1^A(t)]^{n_{59}}}$ | $k_{59} = \beta^{TRR1}_{Cap1} = 6.8863 \times 10^{-11}$<br>$n_{59} = 2$<br>$K_{59} = 7 \times 10^{-8}$ | Fitted<br>Fitted<br>Fitted |
| 60. | $v_{60}(t) = \frac{k_{60} \times [Cap1^A]^{n_{60}}}{(K_{60})^{n_{60}} + [Cap1^A(t)]^{n_{60}}}$ | $k_{60} = \beta^{GSH.mRNA}_{Cap1} = 1.9252 \times 10^{-11}$<br>$n_{60} = 2$<br>$K_{60} = 7 \times 10^{-8}$ | Fitted<br>Fitted<br>Fitted |
| 61. | $v_{61}(t) = \frac{k_{61} \times [Cap1^A]^{n_{61}}}{(K_{61})^{n_{61}} + [Cap1^A(t)]^{n_{61}}}$ | $k_{61} = \beta^{NADPH.mRNA}_{Cap1} = 2.3695 \times 10^{-11}$<br>$n_{61} = 1$<br>$K_{61} = 7 \times 10^{-8}$ | Fitted<br>Fitted<br>Fitted |
| 62. | $v_{62}(t) = \frac{k_{62} \times [Hog1^N.PP(t)]^{n_{62}}}{K_{62}^{n_{62}} + [Hog1^N.PP(t)]^{n_{62}}}$ | $k_{62} = \beta^{CTA1}_{Hog1} = 4.2398 \times 10^{-11}$<br>$n_{62} = 1$<br>$K_{62} = 5 \times 10^{-8}$ | Fitted<br>Fitted<br>Fitted |
| 63. | $v_{63}(t) = k_{63} \times CTA1(t)$ | $k_{63} = r^{CTA1} = 6.4929$ | [5, 4] |
| 64. | $v_{64}(t) = k_{64} \times CAP1(t)$ | $k_{64} = r^{CAP1} = 7.0867 \times 10^{-1}$ | [5, 4] |
| 65. | $v_{65}(t) = k_{65} \times GPX(t)$ | $k_{65} = r^{GPX} = 9.5085 \times 10^{-2}$ | [5, 4] |
| 66. | $v_{66}(t) = k_{66} \times GLR1(t)$ | $k_{66} = r^{GLR1} = 3.1983 \times 10^{-1}$ | [5, 4] |

| | | | |
|---|---|---|---|
| 67. | $v_{67}(t) = k_{67} \times TTR1(t)$ | $k_{67} = r^{TTR1} = 3.2786 \times 10^{-1}$ | [5, 4] |
| 68. | $v_{68}(t) = k_{68} \times TSA1(t)$ | $k_{68} = r^{TSA1} = 4.2782 \times 10^{-1}$ | [5, 4] |
| 69. | $v_{69}(t) = k_{69} \times TRX1(t)$ | $k_{69} = r^{TRX1} = 1.4314 \times 10^{-1}$ | [5, 4] |
| 70. | $v_{70}(t) = k_{70} \times TRR1(t)$ | $k_{70} = r^{TRR1} = 1.0644 \times 10^{1}$ | [5, 4] |
| 71. | $v_{71}(t) = k_{71} \times GSH.mRNA(t)$ | $k_{71} = r^{GSHmRNA} = 1.9626 \times 10^{3}$ | Fitted |
| 72. | $v_{72}(t) = k_{72} \times NADPH.mRNA(t)$ | $k_{72} = r^{NADPHmRNA} = 6.5801 \times 10^{2}$ | Fitted |
| 73. | $v_{73}(t) = k_{73} \times SSK2(t)$ | $k_{73} = r^{SSK2} = 3.7541 \times 10^{-2}$ | [5, 4] |
| 74. | $v_{74}(t) = k_{74} \times PBS2(t)$ | $k_{74} = r^{PBS2} = 6.1608 \times 10^{-1}$ | [5, 4] |
| 75. | $v_{75}(t) = k_{75} \times HOG1(t)$ | $k_{75} = r^{HOG1} = 3.9053 \times 10^{-2}$ | [5, 4] |
| 76. | $v_{76}(t) = \alpha^{H_2O_2} \times H_2O_2^{Ex}(t)$ | $\alpha^{H_2O_2} = 3.8508 \times 10^{-5}$ | Our experimental data. |
| 77. | $v_{77}(t) = \alpha^{H_2O_2} \times H_2O_2^{In}(t)$ | $\alpha^{H_2O_2} = 3.8508 \times 10^{-5}$ | Our experimental data. |
| 78. | $v_{78}(t) = \alpha^{Cat1} \times Cat1(t)$ | $\alpha^{Cat1} = 3.6137 \times 10^{-4}$ | [5] |
| 79. | $v_{79}(t) = \alpha^{GSH} \times GSH(t)$ | $\alpha^{GSH} = 1.2836 \times 10^{-4}$ | [6] |
| 80. | $v_{80}(t) = \alpha^{GSSG} \times GSSG(t)$ | $\alpha^{GSH} = 1.2836 \times 10^{-4}$ | [6] |
| 81. | $v_{81}(t) = \alpha^{Gpx} \times Gpx(t)$ | $\alpha^{Gpx} = 1.4991 \times 10^{-4}$ | [5] |
| 82. | $v_{82}(t) = \alpha^{Glr1} \times Glr1(t)$ | $\alpha^{Glr1} = 3.3007 \times 10^{-4}$ | [5] |
| 83. | $v_{83}(t) = \alpha^{Ttr1} \times Ttr1^{Red}(t)$ | $\alpha^{Ttr1} = 1.0394 \times 10^{-4}$ | [5] |
| 84. | $v_{84}(t) = \alpha^{Ttr1} \times Ttr1^{Ox}(t)$ | $\alpha^{Ttr1} = 1.0394 \times 10^{-4}$ | [5] |
| 85. | $v_{85}(t) = \alpha^{Protein} \times Pr.SH(t)$ | $\alpha^{Protein} = 2.6866 \times 10^{-4}$ | [5] |
| 86. | $v_{86}(t) = \alpha^{Protein} \times Pr.SOH(t)$ | $\alpha^{Protein} = 2.6866 \times 10^{-4}$ | [5] |
| 87. | $v_{87}(t) = \alpha^{Protein} \times Pr.SSG(t)$ | $\alpha^{Protein} = 2.6866 \times 10^{-4}$ | [5] |
| 88. | $v_{88}(t) = \alpha^{Protein} \times Pr.(SH)_2(t)$ | $\alpha^{Protein} = 2.6866 \times 10^{-4}$ | [5] |
| 89. | $v_{89}(t) = \alpha^{Protein} \times Pr.SS(t)$ | $\alpha^{Protein} = 2.6866 \times 10^{-4}$ | [5] |
| 90. | $v_{90}(t) = \alpha^{Tsa1} \times Tsa1^{Red}(t)$ | $\alpha^{Tsa1} = 9.8793 \times 10^{-5}$ | [5] |
| 91. | $v_{91}(t) = \alpha^{Tsa1} \times Tsa1^{Ox}(t)$ | $\alpha^{Tsa1} = 9.8793 \times 10^{-5}$ | [5] |
| 92. | $v_{92}(t) = \alpha^{Trx1} \times Trx1^{Red}(t)$ | $\alpha^{Trx1} = 2.0111 \times 10^{-4}$ | [5] |
| 93. | $v_{93}(t) = \alpha^{Trx1} \times Trx1^{Ox}(t)$ | $\alpha^{Trx1} = 2.0111 \times 10^{-4}$ | [5] |
| 94. | $v_{94}(t) = \alpha^{Trr1} \times Trr1^{Red}(t)$ | $\alpha^{Trr1} = 3.1484 \times 10^{-4}$ | [5] |
| 95. | $v_{95}(t) = \alpha^{Trr1} \times Trr1^{Ox}(t)$ | $\alpha^{Trr1} = 3.1484 \times 10^{-4}$ | [5] |
| 96. | $v_{96}(t) = \alpha^{NADPH} \times NADPH(t)$ | $\alpha^{NADPH} = 1.155 \times 10^{-4}$ | Assumed |

| | | | |
|---|---|---|---|
| 97. | $v_{97}(t) = \alpha^{Cap1} \times Cap1^N(t)$ | $\alpha^{Cap1} = 5.0228 \times 10^{-4}$ | [5] |
| 98. | $v_{98}(t) = \alpha^{Cap1} \times Cap1^A(t)$ | $\alpha^{Cap1} = 5.0228 \times 10^{-4}$ | [5] |
| 99. | $v_{99}(t) = \alpha^{Cap1} \times Cap1^I(t)$ | $\alpha^{Cap1} = 5.0228 \times 10^{-4}$ | [5] |
| 100. | $v_{100}(t) = \alpha^{Ssk2} \times Ssk2(t)$ | $\alpha^{Ssk2} = 2.6866 \times 10^{-4}$ | [5] |
| 101. | $v_{101}(t) = \alpha^{Ssk2} \times Ssk2.P(t)$ | $\alpha^{Ssk2} = 2.6866 \times 10^{-4}$ | [5] |
| 102. | $v_{102}(t) = \alpha^{Pbs2} \times Pbs2(t)$ | $\alpha^{Pbs2} = 3.2090 \times 10^{-4}$ | [5] |
| 103. | $v_{103}(t) = \alpha^{Pbs2} \times Pbs2.PP(t)$ | $\alpha^{Pbs2} = 3.2090 \times 10^{-4}$ | [5] |
| 104. | $v_{104}(t) = \alpha^{Hog1} \times Hog1^N(t)$ | $\alpha^{Hog1} = 4.2317 \times 10^{-5}$ | [5] |
| 105. | $v_{105}(t) = \alpha^{Hog1} \times Hog1^N.PP(t)$ | $\alpha^{Hog1} = 4.2317 \times 10^{-5}$ | [5] |
| 106. | $v_{106}(t) = \alpha^{Hog1} \times Hog1^I(t)$ | $\alpha^{Hog1} = 4.2317 \times 10^{-5}$ | [5] |
| 107. | $v_{107}(t) = \alpha^{Hog1} \times Hog1^I.PP(t)$ | $\alpha^{Hog1} = 4.2317 \times 10^{-5}$ | [5] |
| 108. | $v_{108}(t) = \alpha^{CAT1} \times CAT1(t)$ | $\alpha^{CAT1} = 8.2518 \times 10^{-4}$ | [4] |
| 109. | $v_{109}(t) = \alpha^{CAP1} \times CAP1(t)$ | $\alpha^{CAP1} = 1.0858 \times 10^{-3}$ | [4] |
| 110. | $v_{110}(t) = \alpha^{GPX} \times GPX(t)$ | $\alpha^{GPX} = 8.7358 \times 10^{-4}$ | [4] |
| 111. | $v_{111}(t) = \alpha^{GLR1} \times GLR1(t)$ | $\alpha^{GLR1} = 3.2001 \times 10^{-4}$ | [4] |
| 112. | $v_{112}(t) = \alpha^{TTR1} \times TTR1(t)$ | $\alpha^{TTR1} = 5.4743 \times 10^{-4}$ | [4] |
| 113. | $v_{113}(t) = \alpha^{TSA1} \times TSA1(t)$ | $\alpha^{TSA1} = 4.6448 \times 10^{-4}$ | [4] |
| 114. | $v_{114}(t) = \alpha^{TRX1} \times TRX1(t)$ | $\alpha^{TRX1} = 3.3942 \times 10^{-4}$ | [4] |
| 115. | $v_{115}(t) = \alpha^{TRR1} \times TRR1(t)$ | $\alpha^{TRR1} = 8.0689 \times 10^{-4}$ | [4] |
| 116. | $v_{116}(t) = \alpha^{SSK2} \times SSK2(t)$ | $\alpha^{SSK2} = 1.2097 \times 10^{-3}$ | [4] |
| 117. | $v_{117}(t) = \alpha^{PBS2} \times PBS2(t)$ | $\alpha^{PBS2} = 1.7115 \times 10^{-3}$ | [4] |
| 118. | $v_{118}(t) = \alpha^{HOG1} \times HOG1(t)$ | $\alpha^{HOG1} = 5.3858 \times 10^{-4}$ | [4] |
| 119. | $v_{119}(t) = \alpha^{mRNA} \times GSH.mRNA(t)$ | $\alpha^{mRNA} = 8.2518 \times 10^{-4}$ | [4] |
| 120. | $v_{120}(t) = \alpha^{mRNA} \times NADPH.mRNA(t)$ | $\alpha^{mRNA} = 8.2518 \times 10^{-4}$ | [4] |

The decay rates of the system components were derived from the literature data (with the exception of $H_2O_2^{Ex}$, estimated from our own measurements, and NADPH, assumed). Moreover, we assumed the decay rate of extracellular and intracellular peroxide; GSH and GSSG; as well the various forms of Cap1, Hog1, Tsa1, Trx1, Trr1 to be the same. Similarly, the mRNA synthesis rates were estimated from available literature databases. Since the hypothetical mRNAs represent an entire pathway, their basal rate of synthesis was assumed to be twice the average mRNA synthesis rate. Translation rates were estimated from their corresponding protein degradation rates and mRNA steady state values. Whenever information about the synthesis rate and/or half-life of a particular mRNA or protein was not available, average synthesis and degradation rates were considered to derive the necessary parameter value. Rate constants were taken from the literature when available, and otherwise they were adjusted to fit our experimental data, such as GSH, GSSG, extracellular hydrogen peroxide and mRNA concentrations at the different time points of the measurement.

## References


[1] N. J. Adimora, D. P. Jones, and M. L. Kemp. A model of redox kinetics implicates the thiol proteome in cellular hydrogen peroxide responses. *Antioxidants & Redox Signalling*, 13(6):731–743, 2010.

[2] J. Yu and C-Z. Zhou. Crystal structure of glutathione reductase Glr1 from the yeast *Saccharomyces cerevisiae*. *Proteins: Structure, Function, and Bioinformatics*, 68(4):972–979, 2007.

[3] T. Ohdate, K. Kita, and Y. Inoue. Kinetics and redox regulation of Gpx1, a atypical 2-Cys peroxiredoxin in *Saccharomyces cerevisiae*. *FEMS Yeast Research*, 10(6):787–790, 2010.

[4] C. Miller, B. Schwalb, K. Maier, D. Schulz, S. Dmcke, B. Zacher, A. Mayer, J. Sydow, L. Marcinowski, L. Dlken, D. E. Martin, A. Tresch, and P. Cramer. Dynamic transcriptome analysis measures rates of mrna synthesis and decay in yeast. *Molecular Systems Biology*, 7, 2011.

[5] A. Belle, A. Tanay, L. Bitincka, R. Shamir, and E. K. O'Shea. Quantification of protein half-lives in the budding yeast proteome. *Proceedings of the National Academy of Sciences of the United States of America*, 103(35):13004–13009, 2006.

[6] P Baudouin-Cornu, G Lagniel, C Kumar, M E Huang ME, and J Labarre. Glutathione degradation is a key determinant of glutathione homeostasis. *Journal of Biological Chemistry*, 287(7):4552–45561, 2012.
