# Supplementary material for: Integrative Model of Oxidative Stress Adaptation in the Fungal Pathogen Candida albicans
Source: PLoS One. 2015 Sep 14;10(9):e0137750. doi: 10.1371/journal.pone.0137750 (PMC4569071; doi:10.1371/journal.pone.0137750)
Supplement: S3 Table — (PDF) [file pone.0137750.s006.pdf]

Table S3: List of auxiliary variables used in the oxidative stress response model of *C. albicans*.

No.	Variable	Definition	Description
1.	$S(t)$	$S(t) = \begin{cases} S_1, & \text{if} \quad t = t_1 \\ 0, & \text{if} \quad \text{else.} \end{cases}$	Oxidative stress signal S_1 applied at time t_1.
2.	XS^{In}	$XS^{In}(t) = \begin{cases} H_2O_2^{In}(t) - H_2O_2^{SS}, & \text{if} \quad H_2O_2^{In}(t) - H_2O_2^{SS} > 0 \\ 0, & \text{if} \quad \text{else.} \end{cases}$	Intracellular oxidative stress (XS^{In}). $H_2O_2^{SS} = 1 \times 10^{-9}M$ is steady state value of $H_2O_2^{In}$.
3.	XS^C	$XS^*(t) = \begin{cases} H_2O_2^{In}(t) - H_2O_2^*, & \text{if} \quad H_2O_2^{In}(t) - H_2O_2^* > 0 \\ 0, & \text{if} \quad \text{else.} \end{cases}$	$H_2O_2^*$ is critical threshold value of $H_2O_2^{In}$ that is responsible for the differential regulation of Cap1. Estimated value of $H_2O_2^* = 3.2 \times 10^{-5}M$.
