# Supplementary material for: Integrative Model of Oxidative Stress Adaptation in the Fungal Pathogen Candida albicans
Source: PLoS One. 2015 Sep 14;10(9):e0137750. doi: 10.1371/journal.pone.0137750 (PMC4569071; doi:10.1371/journal.pone.0137750)
Supplement: S4 Table — (PDF) [file pone.0137750.s007.pdf]

Table S4: List of constants used in the oxidative stress response model of *C. albicans*.

No.	Constant	Value	Description
1.	Av	6.023×10^{23}	Avogadro number.
2.	a	1×10^{-3}	Volume conversion factor.
3.	V_t	6.545×10^{-14} L	Total volume of a *C. albicans* cell. Estimated by by assuming that *C. albicans* cells are perfect spheres with radius $r = 5\mu m$.
4.	V_b	2.683×10^{-14} L	Solid base volume of a *C. albicans* cell is 41% [1].
5.	V_{os}	3.862×10^{-14} L	Osmotically active volume, derived from a total cell volume and a solid base volume of 41%.
6.	V_m	2.805×10^{-11} L	Volume of the media is $726.74 \times V_{os}$.
7.	A	7.854×10^{-7} cm^2	Surface area of a *C. albicans* cell.

References

[1] J. Schaber, R. Baltanas, A. Bush, E. Klipp, and A. Colman-Lerner. Modelling reveals novel roles of two parallel signalling pathways and homeostatic feedbacks in yeast. *Molecular Systems Biology*, 8(1), 2012.
