# Supplementary material for: Integrative Model of Oxidative Stress Adaptation in the Fungal Pathogen Candida albicans
Source: PLoS One. 2015 Sep 14;10(9):e0137750. doi: 10.1371/journal.pone.0137750 (PMC4569071; doi:10.1371/journal.pone.0137750)
Supplement: S5 Table — (PDF) [file pone.0137750.s008.pdf]

Table S5: List of ODEs of the oxidative stress response model of *C.albicans*.

No.	ODE
1.	$\frac{d(H_2O_2^{Ex})}{dt} = v_1 + (v_3/V_m) - (v_2/V_m) - v_{76}$
2.	$\frac{d(H_2O_2^{In})}{dt} = v_4 + (v_2/V_{os}) - (v_3/V_{os}) - v_5 - v_8 - v_{10} - v_{15} - v_{18} - v_{77}$
3.	$\frac{d(Cta1)}{dt} = v_{63} - v_{78}$
4.	$\frac{d(GSH)}{dt} = v_{71} + (2 \cdot v_7) - v_6 - (2 \cdot v_8) - v_{11} - v_{13} - v_{79}$
5.	$\frac{d(GSSG)}{dt} = v_6 + v_8 + v_{13} - v_7 - v_{80}$
6.	$\frac{d(Gpx1)}{dt} = v_{65} - v_{81}$
7.	$\frac{d(Glr1)}{dt} = v_{66} - v_{82}$
8.	$\frac{d(Ttr1^{Red})}{dt} = v_{67} + v_{13} - v_{12} - v_{83}$
9.	$\frac{d(Ttr1^{Ox})}{dt} = v_{12} - v_{13} - v_{84}$
10.	$\frac{d(PrSH)}{dt} = v_9 + v_{12} - v_{10} - v_{85}$
11.	$\frac{d(PrSOH)}{dt} = v_{10} - v_{11} - v_{86}$
12.	$\frac{d(PrSSG)}{dt} = v_{11} - v_{12} - v_{87}$
13.	$\frac{d(Pr(SH)_2)}{dt} = v_{14} + v_{16} - v_{15} - v_{88}$
14.	$\frac{d(PrSS)}{dt} = v_{15} - v_{16} - v_{89}$
15.	$\frac{d(Tsa1^{Red})}{dt} = v_{68} + v_{19} - v_{17} - v_{18} - v_{90}$
16.	$\frac{d(Tsa1^{Ox})}{dt} = v_{17} + v_{18} - v_{19} - v_{91}$
17.	$\frac{d(Trx1^{Red})}{dt} = v_{69} + v_{20} - v_{16} - v_{19} - v_{92}$
18.	$\frac{d(Trx1^{Ox})}{dt} = v_{19} + v_{16} - v_{20} - v_{93}$
19.	$\frac{d(Trr1^{Red})}{dt} = v_{70} + v_{21} - v_{20} - v_{94}$
20.	$\frac{d(Trr1^{Ox})}{dt} = v_{20} - v_{21} - v_{95}$
21.	$\frac{d(NADPH)}{dt} = v_{72} + v_{22} - v_7 - v_{21} - v_{96}$

22. $\frac{d(Cap1^N)}{dt} = v_{64} + v_{26} - v_{23} - v_{97}$

23. $\frac{d(Cap1^A)}{dt} = v_{23} + v_{25} - v_{24} - v_{26} - v_{98}$

24. $\frac{d(Cap1^I)}{dt} = v_{24} - v_{25} - v_{99}$

25. $\frac{d(Ssk2)}{dt} = v_{73} + v_{28} - v_{27} - v_{100}$

26. $\frac{d(Ssk2.P)}{dt} = v_{27} - v_{28} - v_{101}$

27. $\frac{d(Pbs2)}{dt} = v_{74} + v_{30} - v_{29} - v_{102}$

28. $\frac{d(Pbs2.PP)}{dt} = v_{29} - v_{30} - v_{103}$

29. $\frac{d(Hog1^N)}{dt} = v_{75} + v_{32} + v_{33} - v_{31} - v_{34} - v_{104}$

30. $\frac{d(Hog1^N.PP)}{dt} = v_{31} + v_{37} - v_{32} - v_{38} - v_{105}$

31. $\frac{d(Hog1^I)}{dt} = v_{34} + v_{36} - v_{33} - v_{35} - v_{106}$

32. $\frac{d(Hog1^I.PP)}{dt} = v_{35} + v_{38} - v_{36} - v_{37} - v_{107}$

33. $\frac{d(CTA1)}{dt} = v_{39} + v_{52} + v_{62} - v_{108}$

34. $\frac{d(CAP1)}{dt} = v_{40} + v_{53} - v_{109}$

35. $\frac{d(GPX)}{dt} = v_{41} + v_{54} - v_{110}$

36. $\frac{d(GLR1)}{dt} = v_{42} + v_{55} - v_{111}$

37. $\frac{d(TTR1)}{dt} = v_{43} + v_{56} - v_{112}$

38. $\frac{d(TSA1)}{dt} = v_{44} + v_{57} - v_{113}$

39. $\frac{d(TRX1)}{dt} = v_{45} + v_{58} - v_{114}$

40. $\frac{d(TRR1)}{dt} = v_{46} + v_{59} - v_{115}$

41. $\frac{d(GSH.mRNA)}{dt} = v_{47} + v_{60} - v_{119}$

42. $\frac{d(NADPH.mRNA)}{dt} = v_{48} + v_{61} - v_{120}$

43. $\frac{d(SSK2)}{dt} = v_{49} - v_{116}$

44. $\frac{d(PBS2)}{dt} = v_{50} - v_{117}$

45. $\frac{d(HOG1)}{dt} = v_{51} - v_{118}$
