# Supplementary material for: Integrative Model of Oxidative Stress Adaptation in the Fungal Pathogen Candida albicans
Source: PLoS One. 2015 Sep 14;10(9):e0137750. doi: 10.1371/journal.pone.0137750 (PMC4569071; doi:10.1371/journal.pone.0137750)
Supplement: S6 Table — (PDF) [file pone.0137750.s009.pdf]

Table S6: List of components of the oxidative stress response model of *C. albicans* and their initial conditions.

No.	Variable	Initial Condition (in M)	Remarks
1.	$H_2O_2^{Ex}$	0	The media is assumed to be free of H_2O_2.
2.	$H_2O_2^{In}$	1×10^{-9}	[1, 2, 3, 4, 5]
3.	$Cat1$	6.4493×10^{-6}	Our experimental data.
4.	GSH	4.708×10^{-3}	Our experimental data.
5.	$GSSG$	2.485×10^{-3}	Our experimental data.
6.	Gpx	1.7284×10^{-7}	[6]
7.	$Glr1$	3.2676×10^{-7}	[6]
8.	$Ttr1^{Red}$	2.5578×10^{-6}	[6]
9.	$Ttr1^{Ox}$	0	At normal conditions, the total no. of oxidised glutaredoxin molecules in *C.albicans* is assumed to be zero.
10.	$Pr.SH$	1.22×10^{-4}	[5]
11.	$Pr.SOH$	0	The concentration of protein sulfenic acid is assumed to be zero in unstressed cells.
12.	$Pr.SSG$	0	The concentration of S-glutathionylated protein mono-thiols is assumed to be zero in unstressed cells.
13.	$Pr.(SH)_2$	1×10^{-3}	[5]
14.	$Pr.SS$	0	The concentration of protein di-sulphides is assumed to be zero during unstressed conditions.
15.	$Tsa1^{Red}$	1.7429×10^{-5}	[6]
16.	$Tsa1^{Ox}$	0	The concentration of oxidised $Tsa1$ is assumed to zero under stress free conditions.
17.	$Trr1^{Red}$	1.2572×10^{-5}	[6]
18.	$Trr1^{Ox}$	0	Under normal condition the concentration of oxidised $Trr1$ proteins is assumed to zero.
19.	$Trx1^{Red}$	1.1518×10^{-6}	[6]
20.	$Trx1^{Ox}$	0	Initial concentration of oxidised form of $Trx1$ is assumed to be zero.
21.	$NADPH$	150×10^{-6}	[7]
22.	$Cap1^{N}$	6.9652×10^{-8}	[6]
23.	$Cap1^{A}$	0	It is assumed that none of $Cap1$ exists in its oxidised active state under normal conditions.
24.	$Cap1^{I}$	0	It is also assumed none of $Cap1$ exists in its oxidised in-active state under normal conditions.
25.	$Ssk2$	1.1738×10^{-8}	[6]
26.	$Ssk2.P$	0	We assume that none of $Ssk2$ exists in its phosphorylated-active form under normal conditions.

27.	$Pbs2$	9.2870×10^{-8}	[6]
28.	$Pbs2.PP$	0	We assume that none of $Pbs2$ exists in its phosphorylated-active form under normal conditions.
29.	$Hog1^N$	2.9151×10^{-7}	[6]
30.	$Hog1^N.PP$	0	It is assumed that none of $Hog1$ exists in its reduced-phosphorylated-inactive form under normal conditions.
31.	$Hog1^I$	0	It is assumed that none of $Hog1$ exists in its oxidised-de-phosphorylated-inactive form under normal conditions.
32.	$Hog1^I.PP$	0	It is assumed that none of $Hog1$ exists in its oxidised-phosphorylated-inactive form under normal conditions.
33.	$CAP1$	4.9367×10^{-11}	[8]
34.	$CTA1$	3.5894×10^{-10}	[8]
35.	GPX	2.7250×10^{-10}	[8]
36.	$GLR1$	3.3723×10^{-10}	[8]
37.	$TTR1$	8.1088×10^{-10}	[8]
38.	$TSA1$	4.0247×10^{-9}	[8]
39.	$TRX1$	1.6183×10^{-9}	[8]
40.	$TRR1$	3.7187×10^{-10}	[8]
41.	$GSH.mRNA$	3.5894×10^{-10}	[8]
42.	$NADPH.mRNA$	3.5894×10^{-10}	[8]
43.	$SSK2$	8.3999×10^{-11}	[8]
44.	$PBS2$	4.8373×10^{-11}	[8]
45.	$HOG1$	3.1587×10^{-10}	[8]

The steady state levels of the intracellular peroxide, various proteins and thiols were taken from the literature. When the data about the initial condition was available in form of molecules/cell, the concentration of the components are estimated by the following formula: $N/(Av \times V_{OS})$, where N is the number of molecules the component present in a single *C. albican* cell, Av is the avogadro number and V_{OS} is osmotically active volume. The steady state levels of the different mRNA molecules were estimated based on literature and our experimental data.

References

[1] N. Oshino, B. Chance, H. Sies, and T. Bcher. The role of H_2O_2 generation in perfused rat liver and the reaction of catalase compound i and hydrogen donors. *Archives of Biochemistry and Biophysics*, 154(1):117–131, 1973.

[2] B. Chance, H. Sies, and A. Boveris. Hydroperoxide metabolism in mammalian organs. *Physiological Reviews*, 59(3):527–605, 1979.

[3] C. Giulivi, P. Hochstein, and K. J. A. Davies. Hydrogen peroxide production by red blood cells. *Free Radical Biology and Medicine*, 16(1):123–129, 1994.

[4] F. Antunes, A. Salvador, H. S. Marinho, R. Alves, and R. E. Pinto. Lipid peroxidation in mitochondrial inner membranes. i. an integrative kinetic model. *Free Radical Biology and Medicine*, 21(7):917–943, 1996.

[5] N. J. Adimora, D. P. Jones, and M. L. Kemp. A model of redox kinetics implicates the thiol proteome in cellular hydrogen peroxide responses. *Antioxidants & Redox Signalling*, 13(6):731–743, 2010.

[6] S. Ghaemmaghami, W. Huh, K. Bower, R. W. Howson, A. Belle, N. Dephoure, E. K. O'Shea, and J. S. Weissman. Global analysis of protein expression in yeast. *Nature*, 425(6959):737–741, 2003.

[7] K. R. Albe, M. H Butler, and B. E. Wright. Cellular concentrations of enzymes and their substrates. *Journal of Theoretical Biology*, 22143(2):163–195, 1990.

[8] C. Miller, B. Schwalb, K. Maier, D. Schulz, S. Dmcke, B. Zacher, A. Mayer, J. Sydow, L. Marcinowski, L. Dlken, D. E. Martin, A. Tresch, and P. Cramer. Dynamic transcriptome analysis measures rates of mrna synthesis and decay in yeast. *Molecular Systems Biology*, 7, 2011.
