# Supplementary material for: Integrative Model of Oxidative Stress Adaptation in the Fungal Pathogen Candida albicans
Source: PLoS One. 2015 Sep 14;10(9):e0137750. doi: 10.1371/journal.pone.0137750 (PMC4569071; doi:10.1371/journal.pone.0137750)
Supplement: S7 Table — (PDF) [file pone.0137750.s010.pdf]

Table S7: *C. albicans* strains used in this study

Strain		Genotype	Source
SC5314	wild type	Clinical isolate	[1]
CAI4	wild type	*ura3::λimm434/ura3::λimm434* (SC5314 lineage)	[2]
RM1000	wild type	*ura3::λimm434/ura3::λimm434, his1::hisG/his1::hisG*	[3]
BWP17	wild type	*ura3::λimm434/ura3::λimm434, his1::hisG/his1::hisG, arg4::hisG/arg4::hisG*	[3]
CA372	wild type	*ura3::λimm434/ura3::λimm434, RPS1-CIp10(URA3)*	[4]
CA674	wild type	*ura3::λimm434/ura3::λimm434, his1::hisG/his1::hisG, RPS1-CIp20(URA3,HIS1)*	[5]
CA1206	wild type	*ura3::λimm434/ura3::λimm434, his1::hisG/his1::hisG, arg4::hisG/arg4::hisG, RPS1-CIp30(URA3,HIS1,ARG4)*	[5]
JC45	*hog1*	*ura3::λimm434/ura3::λimm434, his1::hisG/his1::hisG, hog1::loxP-ura3-loxP/hog1::loxP-HIS1-loxP*	[6]
JC63	Hog1-YFP	*ura3::λimm434/ura3::λimm434, his1hisG/his1hisG, HOG1-YFP-URA3/HOG1-YFP-HIS1*	[7]
JC842	*cap1*	*ura3::λimm434/ura3::λimm434, his1::hisG/his1::hisG, arg4::hisG/arg4::hisG, cap1::loxP-ARG4-loxP/cap1::loxP-HIS1-loxP, RPS1-CIp10(URA3)*	[8]
JC95	Cap1-YFP	*ura3::λimm434/ura3::λimm434, his1::hisG/his1::hisG, CAP1-GFP–URA3*	[7]
JC948	Cap1-MH	*arg4/arg4, leu2/leu2, his1/his1, ura3::λimm434/ura3::λimm434, iro1::λimm434/iro1::λimm434, CAP1-MH–URA3*	[8]
JC118	*cap1 hog1*	*ura3::λimm434/ura3::λimm434, his1::hisG/his1::hisG, arg4::hisG/arg4::hisG, hog1::loxP-ARG4-ura3-loxP/hog1::loxP-HIS1-loxP, cap1::hisG/cap1::hisG-URA3-hisG*	[6]
CA1864	*cat1*	*ura3::λimm434/ura3::λimm434, his1::hisG/his1::hisG, cat1::loxP-URA3-loxP/cat1::HIS1*	[9]
JC677	*trx1*	*trx1::loxP-ARG4-loxP/trx1::loxP- HIS1-loxP, RPS1-CIp10 (URA3)*	[8]
glr1Δ/ glr1Δ	*glr1*	*glr1/glr1, ura3::λimm434/ura3::URA3, his1::hisG/his1::hisG*, *arg4::hisG/arg4::hisG*	[9]

1. **Gillum, A. M., Tsay, E. Y. H. and Kirsch, D. R.** 1984. Isolation of the *Candida albicans* gene for orotidine-5'-phosphate decarboxylase by complementation of *S. cerevisiae ura3* and *Escherichia coli Pyrf* mutations. Molec. General Genet. **198:** 179-182 (1984).
2. **Fonzi, W. A. and Irwin, M. Y.** 1993. Isogenic Strain Construction and Gene-Mapping in *Candida albicans*. Genetics **134:** 717-728.
3. **Wilson, R. B., Davis, D. and Mitchell, A. P.** 1999. Rapid hypothesis testing with *Candida albicans* through gene disruption with short homology regions. J. Bacteriol. **181**: 1868-1874.
4. **Murad, A. M. A., Lee, P. R., Broadbent, I. D., Barelle, C. J. and Brown, A. J. P.** 2000. CIp10, an efficient and convenient integrating vector for *Candida albicans*. Yeast **16**, 325-327.
5. **Dennison, P. M. J., Ramsdale, M., Manson, C. L. and Brown, A. J. P.** 2005. Gene disruption in *Candida albicans* using a synthetic, codon-optimised Cre-loxP system. Fungal Genet. Biol. **42:** 737-748.
6. **Enjalbert, B., D. A. Smith, M. J. Cornell, I. Alam, S. Nicholls, A. J. P. Brown, and J. Quinn.** 2006. Role of the Hog1 stress-activated protein kinase in the global transcriptional response to stress in the fungal pathogen Candida albicans. Mol. Biol. Cell. **17:** 1018-1032.
7. **Smith, D. A., S. Nicholls, B. A. Morgan, A. J. P. Brown, and J. Quinn.** 2004. A conserved stress-activated protein kinase regulates a core stress response in the human pathogen Candida albicans. Mol. Biol. Cell. **15:** 4179-4190.
8. **da Silva Dantas, A., Patterson, M.J., Smith, D.A., MacCallum, D.M., Erwig, L.P., Morgan, B.A. and Quinn, J.** 2010. Thioredoxin regulates multiple hydrogen peroxide-induced signaling pathways in *Candida albicans.* Mol. Cell. Biol. **30:** 4550–4563.
9. **Kaloriti, D., Jacobsen, M., Yin, Z., Patterson, M., Tillmann, A., Smith, D.A., Cook, E., You, T., Grimm, M.J., Bohovych, I., Grebogi, C., Segal, B.H., Gow, N.A.R., Haynes, K., Quinn, J. and Brown, A.J.P.** .2014. Mechanisms underlying the exquisite sensitivity of *Candida albicans* to combinatorial cationic and oxidative stress that enhances the potent fungicidal activity of phagocytes. mBio **5:** e01334-14.
