# Supplementary material for: Integrative Model of Oxidative Stress Adaptation in the Fungal Pathogen Candida albicans
Source: PLoS One. 2015 Sep 14;10(9):e0137750. doi: 10.1371/journal.pone.0137750 (PMC4569071; doi:10.1371/journal.pone.0137750)
Supplement: S8 Table — (PDF) [file pone.0137750.s011.pdf]

Table S8: qRT-PCR primers and probes used in this study.

Gene	Forward primer	Reverse primer
ACT1	ACCACCGGTATTGTTTTGGA	AGCGTAAATTGGAACAACGTG
CAP1	ACCTGAAAAACAAGAGAAAGGTAAAT	GCCGGCACAACTTCATCT
CAT1	TGGTTTTATTCTCCGACAGAGG	TGACCAGAGTAACCATTCATTTCT
TRR1	TTCAGAAACAACCCATTAGCTG	AAATGGCTTCTTCACAAGCTG
